# Exploiting Outage and Error Probability of Cooperative Incremental Relaying in Underwater Wireless Sensor Networks

**DOI:** 10.3390/s16071076

**Published:** 2016-07-12

**Authors:** Hina Nasir, Nadeem Javaid, Muhammad Sher, Umar Qasim, Zahoor Ali Khan, Nabil Alrajeh, Iftikhar Azim Niaz

**Affiliations:** 1 Department of Computer Science, International Islamic University, Islamabad 44000, Pakistan; hinanasir@gmail.com (H.N.); m.sher@iiu.edu.pk (M.S.); 2 COMSATS Institute of Information Technology, Islamabad 44000, Pakistan; ianiaz@comsats.edu.pk; 3 Cameron Library, University of Alberta, Edmonton, AB T6G 2J8, Canada; umar.qasim@ualberta.ca; 4 Internetworking Program, Faculty of Engineering, Dalhousie University, Halifax, NS B3J 4R2, Canada; zahoor.khan@dal.ca; 5 Computer Information Science, Higher Colleges of Technology, Fujairah 4114, United Arab Emirates; 6 Biomedical Technology Department, College of Applied Medical Sciences, King Saud University, Riyadh 11633, Saudi Arabia; nabil@ksu.edu.sa

**Keywords:** underwater wireless sensor network, incremental relaying, cooperative diversity, retransmission, automatic repeat request

## Abstract

This paper embeds a bi-fold contribution for Underwater Wireless Sensor Networks (UWSNs); performance analysis of incremental relaying in terms of outage and error probability, and based on the analysis proposition of two new cooperative routing protocols. Subject to the first contribution, a three step procedure is carried out; a system model is presented, the number of available relays are determined, and based on cooperative incremental retransmission methodology, closed-form expressions for outage and error probability are derived. Subject to the second contribution, Adaptive Cooperation in Energy (ACE) efficient depth based routing and Enhanced-ACE (E-ACE) are presented. In the proposed model, feedback mechanism indicates success or failure of data transmission. If direct transmission is successful, there is no need for relaying by cooperative relay nodes. In case of failure, all the available relays retransmit the data one by one till the desired signal quality is achieved at destination. Simulation results show that the ACE and E-ACE significantly improves network performance, i.e., throughput, when compared with other incremental relaying protocols like Cooperative Automatic Repeat reQuest (CARQ). E-ACE and ACE achieve 69% and 63% more throughput respectively as compared to CARQ in hard underwater environment.

## 1. Introduction

In addition to a wide range of applications, Underwater Wireless Sensor Networks (UWSNs) have gained attention due to significance in emerging paradigms. These networks consist of sensors and vehicles that are deployed in aqueous environment to perform collaborative monitoring tasks. These networks offer variety of applications like assisted navigation, environmental monitoring, resource investigation, tactical surveillance, disaster prevention, etc. [1]. Instead of radio and light waves, UWSNs use acoustic waves due to favourable propagation characteristics in water. Unlike terrestrial WSNs, which use radio and light waves, UWSNs pose some design challenges; long propagation delay, energy constrained sensor nodes, dynamic network topology, huge monitoring area and low bandwidth [1,2,3]. Channel impairments like path loss, multi-path fading, reflection, refraction and aquatic noises introduce high Bit Error Rate (BER) in acoustic transmission and thus lead to lower quality of the received signal [4].

Data critical applications require reliable communication and higher throughput efficiency. Authors in [4,5] suggest that Automatic Repeat reQuest (ARQ) and cooperative diversity schemes efficiently improve the BER. The basic idea of ARQ is to retransmit data in case of failure [4]. On the other hand, Cooperation means sharing each others’s resources to achieve a common goal. Consider a system with a source-destination pair and a relay. Source node broadcasts its data to destination and relay node. As a second replica of the original signal, the relay node forwards the received signal to the destination where both independently received copies are combined to improve quality of the received signal [6].

Cooperative diversity relaying techniques are divided into two categories; fixed relaying and incremental relaying [5]. In fixed relaying, two commonly used techniques are Amplify and Forward (AF) and Decode and Forward (DF). In AF, relay node only amplifies the received signal and forwards it to destination, whereas, in DF, data at relay is decoded, corrected, recoded and forwarded to destination [7]. In incremental relaying, source broadcasts data to destination and relay in a way that feedback is generated from destination about success or failure of the data. In case of negative acknowledgement, relay retransmits data to destination using AF or DF relaying technique, otherwise, source continues with the next packet. Incremental relaying is an on-demand cooperative ARQ scheme and is also termed as Hybrid-ARQ (H-ARQ) scheme [5].

In [8], Ikki et al. present end-to-end performance analysis of incremental relaying cooperative diversity over Rayleigh fading channels, in which the best relay among multiple available relays retransmits the source signal. Similarly, Duy et al. [9] exploit multiple relays and select one of the relays to retransmit signal. However, the aforementioned works only focus on single retransmission (incase of direct transmission failure). In aqueous environment, poor link quality due to severe fading, path-loss and noise may lead to loss of transmitted data. If, somehow, the data reaches its destination, quality is so poor that makes it useless. Therefore, we exploit more number of retransmissions to achieve the desired signal quality at the receiver and require more number of retransmissions. In this regard, we presented closed-form expressions for outage and error probability of incremental relaying with cooperative retransmissions.

Our main contributions in this work are as follows:

1. We proposed incremental relaying cooperative diversity scheme based on multiple number of relays. In our proposed scheme, source broadcasts data to destination and relays. If destination receives an erroneous signal, then relay is responsible to retransmit the signal. Both direct and relayed signals are combined at destination using a diversity combining technique. If signal quality is still not sufficient, second relay is held responsible to retransmit the data. This process continues till either of the two termination criteria are reached; the destination receives a signal with an acceptable quality or all available relays are expired.
2. We proposed two routing protocols for UWSN; ACE [10] and E-ACE. In ACE protocol, retransmission mechanism is incorporated in a cooperative manner to enhance reliability of an existing routing protocol; Energy Efficient Depth Based Routing (EEDBR) [3]. ACE allows only two retransmissions in case of erroneous data reception at destination. However, in noisy underwater environment, two retransmissions may not be sufficient. Therefore, enhanced version of ACE, E-ACE is proposed. In E-ACE, all nodes present in common region of source and destination’s transmission range perform retransmission. Increased number of retransmissions improves reliability and throughput.
3. In our work, the relays perform retransmissions in a cooperative manner. The idea of cooperative retransmissions is taken from CARQ scheme [11]. CARQ scheme is proposed for underwater channel. In this scheme, for a given source-destination pair, neighbor nodes make a cooperative nodes set. The cooperative nodes are selected to retransmit the erroneous packet in a closest one first manner. However, we introduced channel affects and different relay nodes selection criteria in our proposed scheme. For channel affects, BER is calculated at each destination to evaluate the signal quality. Additionally, we presented the cooperative nodes selection method, i.e., minimum depth and highest residual energy among the cooperative nodes. Furthermore, signal combining technique is incorporated in the proposed protocols to reduce Bit Error Rate.

Rest of the paper is organized as follows. Section 2 describes related work, Section 3 presents the proposed schemes and performance analysis in terms of outage and error probability, simulation results and discussions are given in Section 4, and Section 5 concludes the paper.

## 2. Related Work

In [12], authors present a framework for the minimization of collision probability. The problem is considered as a mixed non-linear programming and a mathematical model is developed. Branch and bound algorithm is proposed as a solution of the problem in which optimal number of paths are selected. To reduce the complexity, a near-optimal technique is also proposed. In this technique, the two problems are separated and the problem is reduced to integer non-linear programming. The branch and bound space reduced algorithm is used to solve this problem.

In [13], authors present the dependence of the ergodic capacity and the outage probability of the information transmission to the receiver on the amount of energy transferred to the radio frequency (RF) energy harvesters. A relay selection scheme is proposed which gives the tradeoff between the outage probability and the maximum capacity. Two suboptimal relay selection schemes are also proposed and are applied to the scenarios with limited availability of the channel state information. In this way, the relay node is selected and the performance in terms of throughput and channel capacity is increased.

In [14], authors merged geographic and opportunistic routing. In former routing paradigm, complete path from source to destination is not required. All routing decisions are made locally. In contrast, in opportunistic routing, packet is broadcasted to a set of neighbors. If and only if priority node in the set get failed to forward the packet then other node(s) in set forward the packet. Moreover, authors focused on problems of wireless communication; hidden terminal, void region, and limitation of acoustic channels for underwater environment. For instance, hidden terminal problem, when a node is unable to hear the transmission of other neighbor node then transmissions of both nodes get collide, thus, leading to retransmissions. New enhanced forward set selection algorithm is proposed, which helps to find feasible subset of nodes in which hidden terminal problem is minimized. Beacon algorithm is improved in such a way that a beacon packet has minimum size which avoids overloading of acoustic channels. Furthermore, the other main scientific contribution of this work is recovery mode in void region problem scenario. In this work, authors proposed node’s depth adjustment mechanism to cope with the aforementioned problem.

Authors in [15] exploited the benefit of incremental relaying technique. In this technique, relying is restricted to only worst-case scenario; for example, bad channel conditions. In that case, if outage is declared at destination then it sends acknowledgement to relay(s), in response, relay sends received message to destination either by amplify-and forward or decode-and-forward technique. Authors derived expressions of amplify-and-forward and decode-and-forward technique for Bit Error Rate (BER), outage, and average achievable rate. Extensive simulations and analytical results revealed that incremental relaying technique outperforms direct as well as regular cooperative relaying in terms of BER and average achievable rate.

Energy efficiency in cooperative relaying over fading channels is the main focus of the authors in [16]. A set of selected relay nodes is used to cooperatively beam-form for the data transmission from relay to destination node. In this paper, authors also analyze the total energy cost of data transmission for cooperative beam-forming and acquiring channel state information (CSI). The simulation results confirm that the proposed scheme achieves energy savings up to 16%.

A cross layer design is proposed by the authors in [17]. The Medium Access Control (MAC) and the physical layer power control are combined into the process of node selection. The scheme of minimizing the overall energy consumption and prolonging the network lifetime of cooperative communication is proposed. A set of potential relays is selected on the basis of RTS/CTS (MAC layer) signaling, then the best relay with minimum signaling overhead is selected as a relay. The numerical and simulation results verify the proposed scheme.

In [18], the authors propose a relaying mechanism for the cooperation communication. The new relay mechanism comprises of three things: (1) It identifies relay node’s reputation; (2) It calculates relay node’s SNR; (3) It finds out the location of the particular relay node. Once all of the three parameters are identified, impact of each criterion on relay selection is then calculated with the help of entropy. Shannon’s entropy helps in formulating two vectors; one has the best values of each criterion and the other, worst. These vectors are then utilized by Kullback Leibler and Jensen Shannon divergence to measure dissimilarities between candidates relays nodes and ideal solution which implies that the chosen best relay lowest distance from the ideal solution which is positive and highest distance from the negative ideal solution.

The paper [19] deals with the study performance of two way Amplitude and Forward (AF) cooperative relay networks over Weibull fading channels. This cooperative relaying technique has been discovered to mitigate the effect of fading in wireless networks. The author has derived tight closed form approximations for Overall Outage Probability (OOP) as well as average symbol error probability (ASEP). Parameters such as number of relays, power scaling, power scaling parameters with BPSK and QPSK modulation are varied and then simulated with respect to already calculated OOP and ASEP. The simulated results depict a variety of results such as: ASEP is reduced significantly when the number of relays are increased and so on. OOP and ASEP can be improved by increasing the values of the parameters which include fading severity and power scaling parameters and the overall number of relays.

Hikmat et al. in [20] propose a relay selection technique based on Decode-and-Forward (DF) relaying method. The relay node is selected by using the availability of the Channel State Information (CSI). The authors studied the closed form Symbol Error Rate (SER) for both M Phase Shift Keying (MPSK) and M Quadrature Amplitude Modulation (MQAM) to evaluate decode-and-forward transmission signals. Furthermore, the authors proposed the optimal power method for the relay selection which maximizes the Signal-to-Noise Ratio (SNR) to achieve good performance of the system. Simulation results shows that the SER decreases when the number of relay nodes are increased.

In [21], Sharma et al. studied a joint optimization problem of relay node assignment and flow routing for simultaneous communication sessions. They formulated the problem as a Mixed Integer Linear Programming (MILP) problem and developed a solution based on branch and cut method. The orthogonal channel model is employed in multi-hop wireless network using Amplify-and-Forward (AF) relaying method. The network throughput (rate) is improved by considering Feasible Solution Construction (FSC) for feasible flow of information for cooperative relay node.

Different routing protocols have been designed for underwater sensor networks due to the unique behaviour of underwater acoustic channels. In [22], the authors proposed the underwater opportunistic routing protocol, an opportunistic based routing scheme increases the goodput. The authors focused on two metrics: goodput and energy cost. Goodput is used to measure the amount of useful data received before the deadline and energy cost for per packet energy consumption. In proposed method the authors introduce a new metric EEL success, which is the end to end latency from source to destination when at least one forwarder successfully receives a data. Two step heuristic algorithm designed for forwarding set selection and relay prioritization is also the part of this paper. The opportunistic routing scheme selects the multiple nodes as a forwarding set and let any of those nodes, overhearing the data to forward the data to destination. A data packet is useful only if it arrives at the destination before a certain expiration time.

In [23], the authors design an algorithm to minimize the probability of collision. Cooperative data transmission, optimal allocation of power and selection of route is considered in the proposed algorithm. The relaying technique used is incremental decode and forward. In this technique, the source node transmits its data via direct path, if the destination node is not able to detect the data packet, the relay node forwards the data packet to the destination node. Collision aware routing algorithm is also proposed which minimizes the probability of collision in the network. This algorithm consists of following steps. In first step each node computes the probability of collision when a signal is transmitted from one node to another. In second step, the Bellman-Ford algorithm is used to find the route that causes minimum collision. In third step the optimal power that is calculated by using Langrage Multipliers method is assigned to all nodes.

Energy efficient cooperative routing in terrestrial WSN has been investigated in [24], where N numbers of sensor nodes are randomly deployed in two dimensional (2D) field to monitor a geographical area. The protocol utilizes decode and forward relaying technique to satisfy the prescribed SNR constraint at receiver node. It also utilizes the Bellman-Ford algorithm by assigning the required transmission power as link cost metric and invokes the multi-parametric programming theory to make their proposed framework tractable. This increases the transmission diversity and lowers the transmission power by employing the minimum power relaying policy. However, the trade-off between transmission power and reliability still exists in dense networks.

A clustered WSN is considered in [25] where sensor nodes relay data packets in each cluster to neighbor clusters via cooperative communication. Sensor nodes in the same cluster have very short distance with each other. while the distance between the adjacent clusters is very large as compared to the distance between nodes within the cluster. This scheme functions in two phases: In first Phase nodes in a cluster transmit data packets to the cluster head. Then cluster head (Source node) broadcasts the packet with certain energy to the nodes within the same cluster. In second phase the sensor nodes that decode the packet correctly and the cluster head will transmit the packet to the receiving node. Cluster head is shown that it can adjust its power level for communication within the cluster in order to control the overall number of cooperators such that the overall energy consumption is minimized.

In [26], Energy Efficient Cooperative Communication (EECC) scheme is proposed which is not basically a routing protocol but it helps to minimize packet loss and improves the transmission performance. At each hop between source and sink, cooperative relaying based communication is performed. In case a node does not receive a data packet, nearby nodes which have overheard the packet successfully will cooperate reactively and the best relay is selected out of them to participate in the transmission. This scheme minimizes retransmissions and provides better results in denser networks.

Table 1 shows the comparison of techniques discussed in related work.

## 3. System Performance Analysis and Our Proposed Cooperative Routing Protocols

This section presents outage and error probability performance analysis of incremental relaying cooperative diversity with retransmissions. Based on the analysis, we then propose two cooperative routing protocols; ACE and E-ACE.

### 3.1. System Model and Performance Analysis

**Figure 1:**
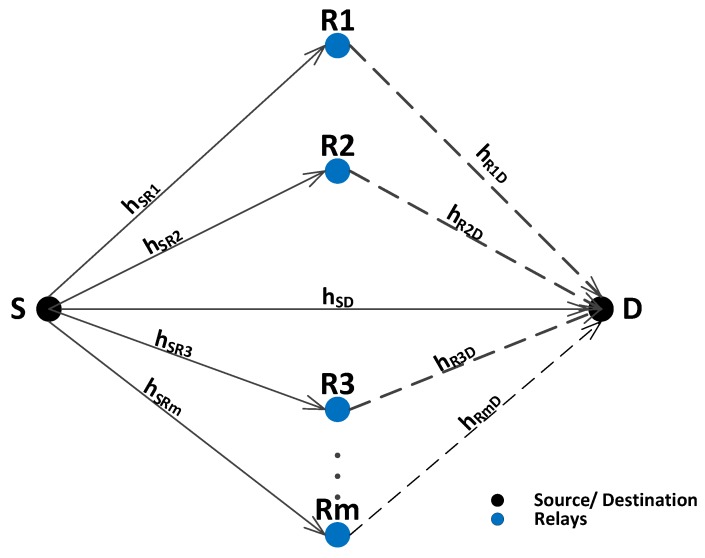
System model.

Figure 1 shows the proposed system model that consists of a Source (S), Destination (D) and Relays (R), where $R=\{R_{1},R_{2},...R_{m}\}$. *m* is maximum number of relays present in the common region of S and D’s transmission range which is also called cooperative region. Each node is equipped with a single omni directional antenna. This work assumes only the special case of non-Line Of Sight (LOS) communication between the nodes due to presence of fish, sea weeds, garbage, salts, etc. For non-LOS communication Rayleigh fading distribution is used. Therefore, we have assumed that each link follows Rayleigh fading as in [27,28]. Additive White Gaussian Noise (AWGN) is also considered to add aquatic noise effect. In the proposed model, Binary Phase Shift Keying (BPSK) is used as a modulation technique, AF as a relaying technique and Maximal Ratio Combining (MRC) as a receive diversity technique.

The proposed system is supposed to follow incremental relaying cooperative diversity. Communication process takes place in two phases. In the first phase, S broadcasts its signal to D and R. If signal received at destination is of sufficient quality, relays are not supposed to retransmit data to destination. On the other hand, if destination receives low quality signal, then relays retransmit the signal one by one till the desired signal quality is achieved at D or all the relays are expired. A relay performs retransmission only once. It is assumed that the channel will experience same fading affects if it performs retransmission multiple times. Therefore, to save energy, relay’s transmission is restricted to single attempt. This assumption also helps to balance the load among other relay nodes. If the retransmission from 1st relay is not successful, it will not retransmit again. Instead, it signals the other available relay to perform retransmission. This process continues till the transmission is successful or the number of available relays are finished.

Quality of a signal is measured in terms of SNR threshold, $\gamma_{0}$. The value of $\gamma_{0}$ depends on the sensing environment. Small value of $\gamma_{0}$ means exclusion of relays and large value of $\gamma_{0}$ means inclusion of relays. The received signals at relays and destination are mathematically expressed as:

(1) $$\begin{array}{c} y_{SD}\left(t\right)=h_{SD}x\left(t\right)+n_{0}\left(t\right) \end{array}$$

(2) $$\begin{array}{c} y_{SR_{i}}\left(t\right)=h_{SR_{i}}x\left(t\right)+n_{j}\left(t\right) \end{array}$$

(3) $$\begin{array}{c} y_{R_{i}D}\left(t\right)=h_{R_{i}D}x_{s}\left(t\right)+n_{i}\left(t\right) \end{array}$$

(4) $$\begin{array}{c} x_{s}\left(t\right)=Gy_{SR_{i}}\left(t\right) \end{array}$$

where i, j = 1→ m. $h_{SD}$, $h_{SR_{i}}$ and $h_{R_{i}D}$ are Rayleigh fading coefficients and $n_{0}$, $n_{i}$ and $n_{j}$ represent channel noises. *x(t)* is transmitted signal at time *t* and $x_{s}\left(t\right)$ is the transmitted signal by the relay at time *t*. $y_{SD}$ represents the signal received at D from S. $y_{SR_{i}}$ is the signal received at $R_{i}$ from S and $y_{R_{i}D}$ is the received signal at D from $R_{i}$.

Since AF relaying technique is used, so, we define G as an amplification factor as:

(5) $$G=\sqrt{\frac{1}{E_{b}h_{SR_{i}}+N_{0}}}$$

where, $N_{0}$ is the power spectral density of AWGN noise and $E_{b}$ is the energy of transmitted signal. Using MRC, the signal at D is given as:

(6) $$y_{D}\left(t\right)=y_{SD}\left(t\right)+y_{R_{i}D}\left(t\right)$$

$y_{D}\left(t\right)$ represents the combined signal of direct and relayed transmission at D. The relays retransmit if and only if the reception at D is erroneous.

### 3.2. Determination of the Number of Available Relays

Relays, present in the cooperative region, retransmit the data whenever direct transmission has SNR less than $\gamma_{0}$. Cooperative region is a common region of S and D’s transmission range. Maximum number of retransmissions depends on the number of relays present in the cooperative region. Thus, for *m* relays, the maximum allowable retransmissions are also *m*.

**Figure 2:**
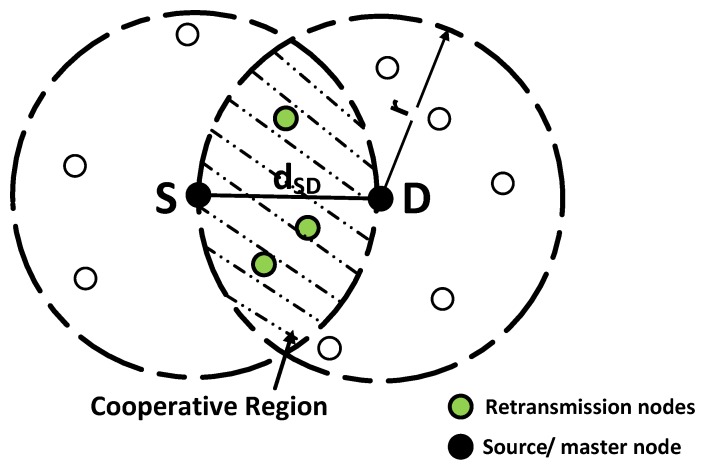
Cooperative region and retransmission nodes.

In this paper, we assume that nodes, *n*, are distributed randomly over an area, *X*, with density, *ρ*. In Figure 2, highlighted region is the overlapping region in which nodes can directly communicate with S and D. In order to find the number of nodes *n* in cooperative region, it is required to find area of cooperative region, defined as *A*. Let the distance between S and D is $d_{SD}$. The node density is given as:

(7) $$\rho =\frac{n}{A}$$

From Appendix A, area of cooperative region with same transmission range, *r*, is calculated as:

(8) $$A=2r^{2}\cos^{-1}\left(\frac{d_{SD}}{2r}\right)-\frac{d_{SD}}{2}\sqrt{4r^{2}-d_{SD}^{2}}$$

Total number of nodes present in cooperative region is ρ × A . Since S and D nodes are also included in that area, therefore, total number of retransmission nodes are given as:

(9) $$\begin{array}{ccc} m & = & \rho \times A\;-2 \end{array}$$

(10) $$\begin{array}{ccc} m & = & \left[\rho \times \left[r^{2}\cos^{-1}\left(\frac{d_{SD}}{2r}\right)-\frac{d_{SD}}{2}\sqrt{4r^{2}-d_{SD}^{2}}\right]\right]-2 \end{array}$$

### 3.3. Outage Probability

Outage is defined as non-availability of signal at D. In the proposed model, outage occurs when direct transmission along with all the retransmissions fail to achieve the desired SNR threshold at D.

The expression for outage probability ($P_{out}$) can be written as:

(11) $$\begin{array}{ccc} P_{out} & = & Pr\left(\gamma_{SD}\leq \gamma_{0}\right)Pr\left(\gamma_{SR_{1}D}+\gamma_{SD}\leq \gamma_{0}|\gamma_{SD}\leq \gamma_{0}\right)\\ & & Pr(\gamma_{SR_{1}D}+\gamma_{SR_{2}D}+\gamma_{SD}\leq \gamma_{0}|\gamma_{SR_{1}D}+\gamma_{SD}\leq \gamma_{0})\cdots \\ & & Pr(\sum_{i=1}^{m}\gamma_{SR_{i}D}+\gamma_{SD}\leq \gamma_{0}|\sum_{i=1}^{m-1}\gamma_{SR_{i}D}+\gamma_{SD}\leq \gamma_{0}) \end{array}$$

In Equation (11), the first term “$Pr(\gamma_{SD}\leq \gamma_{0})$” represents the failure probability of direct link which requires the first relay to retransmit the signal. Therefore, first relay is needed to retransmit the signal. The second term “$Pr(\gamma_{SR_{1}D}+\gamma_{SD}\leq \gamma_{0}|\gamma_{SD}\leq \gamma_{0})$” represents the probability that combined signal at the destination is below $\gamma_{0}$ when direct transmission has already suffered outage. Similarly, the third term “$Pr(\gamma_{SR_{1}D}+\gamma_{SR_{2}D}+\gamma_{SD}\leq \gamma_{0}|\gamma_{SR_{1}D}+\gamma_{SD}\leq \gamma_{0})$” shows that second retransmission is also failed to achieve SNR above $\gamma_{0}$, provided that the first retransmission is also in outage. In third term, second retransmission is combined with the first retransmission along with directly transmitted signal by using MRC. These retransmissions continue till SNR above $\gamma_{0}$ is achieved at destination or all available relays are utilized. When *m*th retransmission fails to achieve SNR greater than $\gamma_{0}$, outage is considered to be occurred. Here $\gamma_{SR_{i}D}$ is the equivalent SNR of $S\to R_{i}\to D$. It is defined in Equation (12) below.

(12) $$\begin{array}{ccc} \gamma_{SR_{i}D} & = & min(\gamma_{SR_{i}},\gamma_{R_{i}D}) \end{array}$$

(13) $$\begin{array}{ccc} & = & \frac{\gamma_{SR_{i}}\gamma_{R_{i}D}}{\gamma_{SR_{i}}+\gamma_{R_{i}D}+1} \end{array}$$

By using law of conditional probability, Equation (11) can be reduced to:

(14) $$P_{out}=Pr\left(\sum_{i=1}^{m}\gamma_{SR_{i}D}+\gamma_{SD}\leq \gamma_{0}\right)$$

In order to calculate a closed-form expression, *m* is limited to 3 for the sake of simplicity. Hence, $P_{out}$ can be expressed as:

(15) $$P_{out}=Pr\left(\gamma_{SR_{1}D}+\gamma_{SR_{2}D}+\gamma_{SR_{3}D}+\gamma_{SD}\leq \gamma_{0}\right)$$

See Appendix C for derivation of Equation (15).

To calculate $P_{out}$, it is required to know the output SNR at the destination. Since MRC is used at destination, the SNR at D, $\gamma_{D}$, is the sum of direct signal, $\gamma_{SD}$, and relayed signals, $\sum_{i=1}^{3}\gamma_{SR_{i}D}$. Where $\gamma_{SR_{i}D}$ is the equivalent SNR of $S\to R_{i}\to D$ [15,29]. A tight upper bound for $\gamma_{SR_{i}D}$ is given in [30]. Since fading is Rayleigh distributed, therefore, *γ* follows exponential distribution with mean $\bar{\gamma }$ [15]. The Probability Distribution Function (PDF) of $min(\gamma_{SR_{i}},\gamma_{R_{i}D})$ is also exponential with a mean ${\bar{\gamma }}_{R_{i}}=min\left({\bar{\gamma }}_{SR_{i}},{\bar{\gamma }}_{R_{i}D}\right)=\frac{{\bar{\gamma }}_{SR_{i}}{\bar{\gamma }}_{R_{i}D}}{{\bar{\gamma }}_{SR_{i}}+{\bar{\gamma }}_{R_{i}D}+1}\;$.

For sum of exponentially distributed independent random variables, their PDF is the convolution of these variables.

The mean of output SNR at D, ${\bar{\gamma }}_{d}$ is given as ${\bar{\gamma }}_{D}={\bar{\gamma }}_{SD}+{\bar{\gamma }}_{R_{1}}+{\bar{\gamma }}_{R_{2}}+{\bar{\gamma }}_{R_{3}}$. PDF of $gamma_{d}$, $f_{\gamma_{d}}$ , is given as:

(16) $$\begin{array}{ccc} f_{\gamma_{d}}\left(z\right) & = & \frac{{\bar{\gamma }}_{SD}^{2}\left[\exp\left(-z/{\bar{\gamma }}_{SD}\right)-\exp\left(-z/{\bar{\gamma }}_{R_{3}}\right)\right]}{\left({\bar{\gamma }}_{SD}-{\bar{\gamma }}_{R_{1}}\right)\left({\bar{\gamma }}_{SD}-{\bar{\gamma }}_{R_{2}}\right)\left({\bar{\gamma }}_{SD}-{\bar{\gamma }}_{R_{3}}\right)}-\\ & & \frac{{\bar{\gamma }}_{SD}{\bar{\gamma }}_{R_{2}}\left[\exp\left(-z/{\bar{\gamma }}_{R_{2}}\right)-\exp\left(-z/{\bar{\gamma }}_{R_{3}}\right)\right]}{\left({\bar{\gamma }}_{SD}-{\bar{\gamma }}_{R_{1}}\right)\left({\bar{\gamma }}_{SD}-{\bar{\gamma }}_{R_{2}}\right)\left({\bar{\gamma }}_{R_{2}}-{\bar{\gamma }}_{R_{3}}\right)}+\\ & & \frac{{\bar{\gamma }}_{R_{1}}\left[\upsilon +\tau \right]}{\left({\bar{\gamma }}_{SD}-{\bar{\gamma }}_{R_{1}}\right)\left({\bar{\gamma }}_{R_{1}}-{\bar{\gamma }}_{R_{2}}\right)} \end{array}$$

where,

(17) $$\upsilon =\frac{{\bar{\gamma }}_{R_{2}}\left[\exp\left(-z/{\bar{\gamma }}_{R_{2}}\right)-\exp\left(-z/{\bar{\gamma }}_{R_{3}}\right)\right]}{{\bar{\gamma }}_{R_{2}}-{\bar{\gamma }}_{R_{3}}}$$

and

(18) $$\tau =\frac{{\bar{\gamma }}_{R_{1}}\left[\exp\left(-z/{\bar{\gamma }}_{R_{1}}\right)-\exp\left(-z/{\bar{\gamma }}_{R_{3}}\right)\right]}{{\bar{\gamma }}_{R_{1}}-{\bar{\gamma }}_{R_{3}}}$$

The detailed derivation of $f_{\gamma_{d}}$ is given in Appendix B.

By integrating Equation (B6) and doing some necessary simplification, we get closed-form expression for $P_{out}$ as:

(19) $$\begin{array}{ccc} P_{out} & = & 1+\frac{{\bar{\gamma }}_{SD}^{2}\left({\bar{\gamma }}_{R_{3}}exp\left(-\gamma_{0}/{\bar{\gamma }}_{R_{3}}\right)-{\bar{\gamma }}_{SD}exp\left(-\gamma_{0}/{\bar{\gamma }}_{SD}\right)\right)}{\left({\bar{\gamma }}_{SD}-{\bar{\gamma }}_{R_{2}}\right)\left({\bar{\gamma }}_{SD}-{\bar{\gamma }}_{R_{3}}\right)\left({\bar{\gamma }}_{SD}-{\bar{\gamma }}_{R_{1}}\right)}+\\ & & \frac{{\bar{\gamma }}_{R_{2}}\left({\bar{\gamma }}_{R_{2}}exp\left(-\gamma_{0}/{\bar{\gamma }}_{R_{2}}\right)-{\bar{\gamma }}_{R_{3}}exp\left(-\gamma_{0}/{\bar{\gamma }}_{R_{3}}\right)\right)}{\left({\bar{\gamma }}_{SD}-{\bar{\gamma }}_{R_{2}}\right)\left({\bar{\gamma }}_{R_{2}}-{\bar{\gamma }}_{R_{3}}\right)\left({\bar{\gamma }}_{SD}-{\bar{\gamma }}_{R_{1}}\right)}+\\ & & \frac{{\bar{\gamma }}_{R_{1}}{\bar{\gamma }}_{R_{2}}\left({\bar{\gamma }}_{R_{3}}exp\left(-\gamma_{0}/\bar{\gamma_{R_{3}}}\right)-{\bar{\gamma }}_{R_{2}}exp\left(-\gamma_{0}/\bar{\gamma_{R_{2}}}\right)\right)}{\left({\bar{\gamma }}_{R_{2}}-{\bar{\gamma }}_{R_{3}}\right)\left({\bar{\gamma }}_{R_{1}}-{\bar{\gamma }}_{R_{2}}\right)\left({\bar{\gamma }}_{SD}-{\bar{\gamma }}_{R_{1}}\right)}+\\ & & \frac{{\bar{\gamma }}_{R_{1}}^{2}\left({\bar{\gamma }}_{R_{1}}exp\left(-\gamma_{0}/\bar{\gamma_{R_{1}}}\right)-{\bar{\gamma }}_{R_{3}}exp\left(-\gamma_{0}/\bar{\gamma_{R_{3}}}\right)\right)}{\left(\bar{\gamma_{R_{1}}}-\bar{\gamma_{R_{3}}}\right)\left(\bar{\gamma_{R_{1}}}-\bar{\gamma_{R_{2}}}\right)\left(\bar{\gamma_{SD}}-\bar{\gamma_{R_{1}}}\right)} \end{array}$$

**Figure 3:**
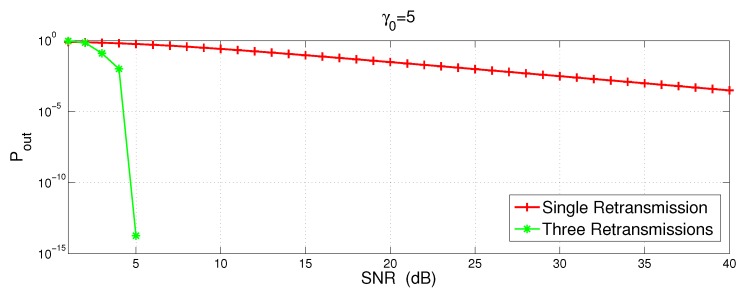
$P_{out}$ of incremental relaying with cooperative retransmissions.

Figure 3 shows the outage probability with one and three retransmissions vs. SNR of the transmitted signal. The SNR is varied from 1 to 40 dB. This simulation is conducted for the case when $\gamma_{SD}\neq \gamma_{R_{1}}\neq \gamma_{R_{2}}\neq \gamma_{R_{3}}$ and $\gamma_{0}$ is set to 5 dB. Figure 3 clearly shows that more number of retransmissions reduces the outage probability. With three retransmission, the transmitted signal with low SNR, i.e., 5dB, outage probability is $10^{-15}$. This is because, in case of error after first retransmission, second retransmission may help the system to get out of outage and same is the case with third retransmission.

### 3.4. Error Probability

In this section, closed-form expressions for error probability of incremental relaying with cooperative retransmissions has been derived for AF relaying. The average un-conditional error probability, P(e) is given as [15]:

(20) $$P\left(e\right)=Pr\left(\gamma_{SD}\leq \gamma_{0}\right)\times P_{div}\left(e\right)+\left(1-Pr\left(\gamma_{SD}\leq \gamma_{0}\right)\right)\times P_{direct}\left(e\right)$$

The first term, $Pr\left(\gamma_{SD}\leq \gamma_{0}\right)\times P_{div}\left(e\right)$, in the above equation represents that the error occurs in the direct transmission and assistance from relays is needed. The fading is Rayleigh distributed, therefore, $\gamma_{SD}$ follows exponential distribution. Hence $Pr(\gamma_{SD})$ is written as:

(21) $$Pr\left(\gamma_{SD}\leq \gamma_{0}\right)=1-exp\left(-\frac{\gamma_{0}}{{\bar{\gamma }}_{SD}}\right)$$

Here $\gamma_{SD}$ is the SNR between S and D. $P_{div}\left(e\right)$ in Equation (20) represents the average probability that an error occurs in the combined diversity transmission from S and R to the D. $P_{direct}\left(e\right)$ represents the probability of error at D given that direct transmission is successful and relay’s assistance is not required. The conditional error probability takes the form of $a\times erfc(\sqrt{b\gamma_{SD}})$, with erfc(x) is the error function defines as $erfc\left(x\right)=\left(2/\pi \right)\int_{0}^{\infty }exp\left(-x^{2}\right)\;dx)$ and (a,b) are constants. For BPSK: a = 0.5 and b = 1. Hence, $P_{direct}\left(e\right)$ can be written as

(22) $$P_{direct}\left(e\right)=\int_{0}^{\infty }a\times erfc\left(\sqrt{b\gamma }\right)f_{\gamma_{SD}}\left(\gamma |\gamma_{SD}>\gamma_{0}\right)\;d\gamma$$

where, $f_{\gamma_{SD}}\left(\gamma |\gamma_{SD}>\gamma_{0}\right)$ is the conditional PDF of $\gamma_{SD}$ given that $\gamma_{SD}$ is greater than the threshold $\gamma_{0}$.

The conditional PDF, $f_{\gamma_{SD}}\left(\gamma |\gamma_{SD}>\gamma_{0}\right)$, for $\gamma >\gamma_{0}$ is found as:

(23) $$f_{\gamma_{SD}}\left(\gamma |\gamma_{SD}>\gamma_{0}\right)=\frac{exp(\gamma_{0}/{\bar{\gamma }}_{SD})}{{\bar{\gamma }}_{SD}}exp\left(\frac{-\bar{\gamma }}{{\bar{\gamma }}_{SD}}\right)$$

The closed-form expression for $P_{direct}\left(e\right)$, after substituting Equation (23) into Equation (22), solving the integration and doing some required manipulations, is written as:

(24) $$P_{direct}\left(e\right)=a\;erfc\left(\sqrt{b\gamma_{0}}\right)-a\;exp\left(\frac{\gamma_{0}}{{\bar{\gamma }}_{SD}}\right)\times \sqrt{\frac{b{\bar{\gamma }}_{SD}}{1+b{\bar{\gamma }}_{SD}}}\;erfc\left(\sqrt{\gamma_{0}(b+1/{\bar{\gamma }}_{SD}}\right))$$

If the destination needs assistance from relays, the signals are combined at D using MRC. To calculate $P_{div}\left(e\right)$, we need to know the output SNR at D. The output SNR at D will be sum of direct signal, $\gamma_{SD}$ and relayed signals. For incremental relaying cooperative retransmissions, $P_{div}\left(e\right)$ will have three cases.

**Case 1**: $\left(\gamma_{SD}\leq \gamma_{0}\right)$
  In this case, the relay, $R_{1}$, performs re-transmission given that direct transmission is not successful. In this case, $\gamma_{d}=\gamma_{SD}+\gamma_{R_{1}}$, where $\gamma_{d}$ represents output SNR at D and $\gamma_{R_{i}}$ is defined in Equation (B2). $P_{div}\left(e\right)$ can be written as
  (25) $$P_{div}\left(e\right)=\int_{0}^{\infty }a\times erfc\left(\sqrt{bz}\right)f_{\gamma_{d}}\left(z|\gamma_{SD}\leq \gamma_{0}\right)\;dz$$
  $\gamma_{d}$ is a random variable whose PDF is the convolution of the PDFs of $\gamma_{SD}$ and $\gamma_{R_{1}}=f_{\gamma_{1}}$. $f_{\gamma_{1}}$ is derived in Appendix B in Equation (B3). The conditional PDF of output SNR at D, $f_{\gamma_{d}}\left(z|\gamma_{SD}\;\leq \;\gamma_{0}\right)$ is derived as:
  (26) $$\begin{array}{ccc} f_{\gamma_{d}}\left(z|\gamma_{SD}\leq \gamma_{0}\right) & = & \frac{f_{\gamma_{SD}}\left(z\right)*f_{\gamma_{R_{1}}}\left(z\right)}{Pr[\gamma_{SD}\leq \gamma_{0}]}\\ & = & \frac{exp\left(-z/{\bar{\gamma }}_{R_{1}}\right)-exp\left(-z/{\bar{\gamma }}_{SD}\right)}{\left({\bar{\gamma }}_{R_{1}}-{\bar{\gamma }}_{SD}\right)\left(1-exp\left(-\gamma_{0}/{\bar{\gamma }}_{SD}\right)\right)},\;\;\;\;\;\;\;\;\;\;\;\;\;\;\;\;if\;\;z\leq \gamma_{0} \end{array}$$
  where, $Pr[\gamma_{SD}\leq \gamma_{0}]$ is defined in Equation (21). Now, the closed-form expression for $P_{div}\left(e\right)$ is obtained by putting Equation (26) in Equation (25) and performing integration. We need to integrate from 0 to $\gamma_{0}$ for case $\gamma_{d}\leq \gamma_{0}$.
  (27) $$\begin{array}{c} \begin{array}{ccc} P_{div}\left(e\right) & = & \frac{a}{\left({\bar{\gamma }}_{R_{1}}-{\bar{\gamma }}_{SD}\right)\left(1-e^{\frac{-\gamma_{0}}{{\bar{\gamma }}_{SD}}}\right)}\times \\ & & \left[{\bar{\gamma }}_{R_{1}}-{\bar{\gamma }}_{R_{1}}\sqrt{b{\bar{\gamma }}_{R_{1}}\left(1+b{\bar{\gamma }}_{R_{1}}\right)}erf\left(\sqrt{\gamma_{0}\left(b+1/{\bar{\gamma }}_{R_{1}}\right)}\right)-{\bar{\gamma }}_{R_{1}}e^{\frac{-\gamma_{0}}{{\bar{\gamma }}_{R_{1}}}}erfc\left(\sqrt{\gamma_{0}b}\right)\right]-\\ & & \frac{a}{\left(-{\bar{\gamma }}_{R_{1}}+{\bar{\gamma }}_{SD}\right)\left(-1+e^{\frac{-\gamma_{0}}{{\bar{\gamma }}_{SD}}}\right)}\times \\ & & {\bar{\gamma }}_{SD}\left[-e^{\frac{-\gamma_{0}}{{\bar{\gamma }}_{SD}}}+\sqrt{b{\bar{\gamma }}_{SD}\left(1+b{\bar{\gamma }}_{SD}\right)}e^{\frac{-\gamma_{0}}{{\bar{\gamma }}_{SD}}}erf\left(\sqrt{\gamma_{0}\left(b+1/{\bar{\gamma }}_{SD}\right)}\right)+erfc\left(\sqrt{\gamma_{0}b}\right)\right] \end{array} \end{array}$$
**Case 2**: $\left[\left(\gamma_{SD}+\gamma_{R_{1}}\right)\leq \gamma_{0}\right]$
  In this case, relays, $R_{1}$ and $R_{2}$, perform retransmission given that first retransmission was not successful. Here, $\gamma_{d}=\gamma_{SD}+\gamma_{R_{1}}+\gamma_{R_{2}}$, where $\gamma_{d}$ represents output SNR at D and $\gamma_{R_{i}}$ is given in Equation (B2). $P_{div}\left(e\right)$ can be written as:
  (28) $$P_{div}\left(e\right)=\int_{0}^{\infty }a\times erfc\left(\sqrt{bz}\right)f_{\gamma_{d}}\left(z|\left(\gamma_{SD}+\gamma_{R_{1}}\right)\leq \gamma_{0}\right)\;dz$$
  The PDF of random variable, $\gamma_{d}$, will be the convolution of the PDFs of $\gamma_{SD}$, $\gamma_{R_{1}}$ and $\gamma_{R_{2}}$. Hence, $f_{\gamma_{d}}\left(z|\left(\gamma_{SD}+\gamma_{R_{1}}\right)\leq \gamma_{0}\right)$ for case $z\leq \gamma_{0}$ is derived as:
  (29) $$\begin{array}{ccc} f_{\gamma_{d}}\left(z|\left(\gamma_{SD}+\gamma_{R_{1}}\right)\leq \gamma_{0}\right) & = & \frac{f_{\gamma_{SD}}\left(z\right)*f_{\gamma_{R_{1}}}\left(z\right)*f_{\gamma_{R_{2}}}\left(z\right)}{Pr[\gamma_{SD}+\gamma_{R_{1}}\leq \gamma_{0}]}\\ & = & \frac{1}{\left({\bar{\gamma }}_{SD}\left(-e^{\frac{-\gamma_{0}}{{\bar{\gamma }}_{SD}}}+1\right)-{\bar{\gamma }}_{R_{1}}\left(-e^{\frac{-\gamma_{0}}{{\bar{\gamma }}_{R_{1}}}}+1\right)\right)}\times \\ & & \left[\frac{{\bar{\gamma }}_{SD}}{{\bar{\gamma }}_{SD}-{\bar{\gamma }}_{R_{2}}}\left[exp\left(-z/{\bar{\gamma }}_{SD}\right)-exp\left(-z/{\bar{\gamma }}_{R_{2}}\right)\right]-\right.\\ & & \left.\frac{{\bar{\gamma }}_{R_{1}}}{{\bar{\gamma }}_{R_{1}}-{\bar{\gamma }}_{R_{2}}}\left[exp\left(-z/{\bar{\gamma }}_{R_{1}}\right)-exp\left(-z/{\bar{\gamma }}_{R_{2}}\right)\right]\right] \end{array}$$
  where $f_{\gamma_{SD}}\left(z\right)*f_{\gamma_{R_{1}}}\left(z\right)*f_{\gamma_{R_{2}}}\left(z\right)=f_{\gamma_{2}}$. $f_{\gamma_{2}}$ is defined in Appendix B in Equation (B4). $Pr[\gamma_{SD}+\gamma_{R_{1}}\leq \gamma_{0}]$ is obtained by integrating Equation (B3) from 0 to $\gamma_{0}$.
  Substituting Equation (29) in Equation (28), integrating and doing necessary manipulations, the closed-form expression for $P_{div}\left(e\right)$ is given as:
  (30) $$\begin{array}{ccc} P_{div}\left(e\right) & = & \frac{a}{{\bar{\gamma }}_{SD}\left(1-\exp\left(-\gamma_{0}/{\bar{\gamma }}_{SD}\right)\right)+{\bar{\gamma }}_{R_{1}}\left(-1+\exp\left(-\gamma_{0}/{\bar{\gamma }}_{R_{1}}\right)\right)}\times \\ & & \left[\frac{{\bar{\gamma }}_{SD}^{2}\left(1-e^{\frac{-\gamma_{0}}{{\bar{\gamma }}_{SD}}}+e^{\frac{-\gamma_{0}}{{\bar{\gamma }}_{SD}}}erf\left(\sqrt{b\gamma_{0}}\right)-\sqrt{u}\;erf\left(\sqrt{\gamma_{0}\left(b+1/{\bar{\gamma }}_{SD}\right)}\right)\right)}{{\bar{\gamma }}_{SD}-{\bar{\gamma }}_{R_{2}}}-\right.\\ & & \left.\frac{{\bar{\gamma }}_{R_{1}}^{2}\left(1-e^{\frac{-\gamma_{0}}{{\bar{\gamma }}_{R_{1}}}}+e^{\frac{-\gamma_{0}}{{\bar{\gamma }}_{R_{1}}}}erf\left(\sqrt{b\gamma_{0}}\right)-\sqrt{v}\;erf\left(\sqrt{\gamma_{0}\left(b+1/{\bar{\gamma }}_{R_{1}}\right)}\right)\right)}{{\bar{\gamma }}_{R_{1}}-{\bar{\gamma }}_{R_{2}}}-\right.\\ & & \left.\frac{\gamma_{SD}\gamma_{R_{2}}\left(1-e^{\frac{-\gamma_{0}}{{\bar{\gamma }}_{R_{2}}}}+e^{\frac{-\gamma_{0}}{{\bar{\gamma }}_{R_{2}}}}erf\left(\sqrt{b\gamma_{0}}\right)-\sqrt{w}\;erf\left(\sqrt{\gamma_{0}\left(b+1/{\bar{\gamma }}_{R_{2}}\right)}\right)\right)}{{\bar{\gamma }}_{SD}-{\bar{\gamma }}_{R_{2}}}+\right.\\ & & \left.\frac{{\bar{\gamma }}_{R_{1}}{\bar{\gamma }}_{R_{2}}\left(1-e^{\frac{-\gamma_{0}}{{\bar{\gamma }}_{R_{2}}}}+e^{\frac{-\gamma_{0}}{{\bar{\gamma }}_{R_{2}}}}erf\left(\sqrt{b\gamma_{0}}\right)-\sqrt{w}\;erf\left(\sqrt{\gamma_{0}\left(b+1/{\bar{\gamma }}_{R_{2}}\right)}\right)\right)}{{\bar{\gamma }}_{R_{1}}-{\bar{\gamma }}_{R_{2}}}\right] \end{array}$$
  where,
  (31) $$u=\frac{{\bar{\gamma }}_{SD}b}{1+{\bar{\gamma }}_{SD}b}$$
  (32) $$v=\frac{{\bar{\gamma }}_{R_{1}}b}{1+{\bar{\gamma }}_{R_{1}}b}$$
  (33) $$w=\frac{{\bar{\gamma }}_{R_{2}}b}{1+{\bar{\gamma }}_{R_{2}}b}$$
**Case 3**: $\left[\left(\gamma_{SD}+\gamma_{R_{1}}+\gamma_{R_{2}}\right)\leq \gamma_{0}\right]$
  For this case, relays, $R_{1},R_{2}$ and $R_{3}$, perform retransmission given that second retransmission was not successful and third retransmission is required from $R_{3}$. Here, $\gamma_{d}=\gamma_{SD}+\gamma_{R_{1}}+\gamma_{R_{2}}\;+\;\gamma_{R_{2}}$, where $\gamma_{d}$ represents output SNR at D and $\gamma_{R_{i}}$ is from Equation (B2). $P_{div}\left(e\right)$ can be written as
  (34) $$P_{div}\left(e\right)=\int_{0}^{\infty }a\times erfc\left(\sqrt{bz}\right)f_{\gamma_{d}}\left(z|\left(\gamma_{SD}+\gamma_{R_{1}}+\gamma_{R_{2}}\right)\leq \gamma_{0}\right)\;dz$$
  The PDF of random variable, $\gamma_{d}$, in this case will be the convolution of the PDFs of $\gamma_{SD}$, $\gamma_{R_{1}}$, $\gamma_{R_{2}}$ and $\gamma_{R_{3}}$. So, $f_{\gamma_{d}}\left(z|\left(\gamma_{SD}+\gamma_{R_{1}}+\gamma_{R_{2}}\right)\leq \gamma_{0}\right)$ for case $z\leq \gamma_{0}$ is given by:
  (35) $$\begin{array}{ccc} f_{\gamma_{d}}\left(z|\left(\gamma_{SD}+\gamma_{R_{1}}+\gamma_{R_{2}}\right)\leq \gamma_{0}\right) & = & \frac{f_{\gamma_{SD}}\left(z\right)*f_{\gamma_{R_{1}}}\left(z\right)*f_{\gamma_{R_{2}}}\left(z\right)*f_{\gamma_{R_{3}}}}{Pr[\gamma_{SD}+\gamma_{R_{1}}+\gamma_{R_{2}}\leq \gamma_{0}]}\\ & = & \left[\frac{{\bar{\gamma }}_{SD}^{2}\left[\exp\left(-z/{\bar{\gamma }}_{SD}\right)-\exp\left(-z/{\bar{\gamma }}_{R_{3}}\right)\right]}{\left({\bar{\gamma }}_{SD}-{\bar{\gamma }}_{R_{2}}\right)\left({\bar{\gamma }}_{SD}-{\bar{\gamma }}_{R_{3}}\right)}-\right.\\ & & \left.\frac{{\bar{\gamma }}_{SD}\times \upsilon }{\left({\bar{\gamma }}_{R_{2}}-{\bar{\gamma }}_{R_{3}}\right)}+\frac{{\bar{\gamma }}_{R_{1}}\left[\upsilon +\tau \right]}{\left({\bar{\gamma }}_{R_{1}}-{\bar{\gamma }}_{R_{2}}\right)}\right]÷\\ & & \left[\frac{{\bar{\gamma }}_{SD}^{2}\left(1-\exp\left(-\gamma_{0}/{\bar{\gamma }}_{SD}\right)\right)}{{\bar{\gamma }}_{SD}-{\bar{\gamma }}_{R_{2}}}-\right.\\ & & \left.\frac{{\bar{\gamma }}_{SD}{\bar{\gamma }}_{R_{2}}\left(1-\exp\left(-\gamma_{0}/{\bar{\gamma }}_{R_{2}}\right)\right)}{{\bar{\gamma }}_{SD}-{\bar{\gamma }}_{R_{2}}}-\right.\\ & & \left.\frac{{\bar{\gamma }}_{R_{1}}\left({\bar{\gamma }}_{R_{1}}-{\bar{\gamma }}_{R_{1}}e^{\frac{-\gamma_{0}}{{\bar{\gamma }}_{R_{1}}}}+{\bar{\gamma }}_{R_{2}}\left(-1+e^{\frac{-\gamma_{0}}{{\bar{\gamma }}_{R_{2}}}}\right)\right)}{{\bar{\gamma }}_{R_{1}}-{\bar{\gamma }}_{R_{2}}}\right] \end{array}$$
  where:
  (36) $$\upsilon =\frac{{\bar{\gamma }}_{R_{2}}\left[\exp\left(-z/{\bar{\gamma }}_{R_{2}}\right)-\exp\left(-z/{\bar{\gamma }}_{R_{3}}\right)\right]}{{\bar{\gamma }}_{R_{2}}-{\bar{\gamma }}_{R_{3}}}$$
  and
  (37) $$\tau =\frac{{\bar{\gamma }}_{R_{1}}\left[\exp\left(-z/{\bar{\gamma }}_{R_{1}}\right)-\exp\left(-z/{\bar{\gamma }}_{R_{3}}\right)\right]}{{\bar{\gamma }}_{R_{1}}-{\bar{\gamma }}_{R_{3}}}$$
  $f_{\gamma_{SD}}\left(z\right)*f_{\gamma_{R_{1}}}\left(z\right)*f_{\gamma_{R_{2}}}\left(z\right)*f_{\gamma_{R_{3}}}=f_{\gamma_{3}}$. $f_{\gamma_{3}}$ is derived in Equation (B5) in Appendix B and $Pr[\gamma_{SD}+\gamma_{R_{1}}+\gamma_{R_{2}}\leq \gamma_{0}]$ is obtained by integration Equation (B4) from 0 to $\gamma_{0}$. By substituting the above equation in Equation (34) and integrating, we get closed-form expression for $P_{div}\left(e\right)$ as:
  (38) $$\begin{array}{ccc} P_{div}\left(e\right) & = & \frac{a}{\frac{{\bar{\gamma }}_{SD}^{2}\left(1-e^{\frac{-\gamma_{0}}{{\bar{\gamma }}_{SD}}}\right)}{{\bar{\gamma }}_{SD}-{\bar{\gamma }}_{R_{2}}}-\frac{{\bar{\gamma }}_{SD}{\bar{\gamma }}_{R_{2}}\left(1-e^{\frac{-\gamma_{0}}{{\bar{\gamma }}_{R_{2}}}}\right)}{{\bar{\gamma }}_{SD}-{\bar{\gamma }}_{R_{2}}}-\frac{{\bar{\gamma }}_{R_{1}}\left({\bar{\gamma }}_{R_{1}}-{\bar{\gamma }}_{R_{1}}e^{\frac{-\gamma_{0}}{{\bar{\gamma }}_{R_{1}}}}+{\bar{\gamma }}_{R_{2}}\left(-1+e^{\frac{-\gamma_{0}}{{\bar{\gamma }}_{R_{2}}}}\right)\right)}{{\bar{\gamma }}_{R_{1}}-{\bar{\gamma }}_{R_{2}}}}\\ & & \left[\frac{{\bar{\gamma }}_{SD}^{3}\left(1-e^{\frac{-\gamma_{0}}{{\bar{\gamma }}_{SD}}}+e^{\frac{-\gamma_{0}}{{\bar{\gamma }}_{SD}}}erf\left(\sqrt{b\gamma_{0}}\right)-\sqrt{\frac{{\bar{\gamma }}_{SD}b}{1+{\bar{\gamma }}_{SD}b}}erf\left(\sqrt{\gamma_{0}\left(b+1/{\bar{\gamma }}_{SD}\right)}\right)\right)}{\left({\bar{\gamma }}_{SD}-{\bar{\gamma }}_{R_{2}}\right)\left({\bar{\gamma }}_{SD}-{\bar{\gamma }}_{R_{3}}\right)}-\right.\\ & & \left.\frac{{\bar{\gamma }}_{R_{1}}^{3}\left(1-e^{\frac{-\gamma_{0}}{{\bar{\gamma }}_{R_{1}}}}+e^{\frac{-\gamma_{0}}{{\bar{\gamma }}_{R_{1}}}}erf\left(\sqrt{b\gamma_{0}}\right)-\sqrt{\frac{{\bar{\gamma }}_{R_{1}}b}{1+{\bar{\gamma }}_{R_{1}}b}}erf\left(\sqrt{\gamma_{0}\left(b+1/{\bar{\gamma }}_{R_{1}}\right)}\right)\right)}{\left({\bar{\gamma }}_{R_{1}}-{\bar{\gamma }}_{R_{2}}\right)\left({\bar{\gamma }}_{R_{1}}-{\bar{\gamma }}_{R_{2}}\right)}-\right.\\ & & \left.\frac{{\bar{\gamma }}_{SD}{\bar{\gamma }}_{R_{2}}^{2}\left(1-e^{\frac{-\gamma_{0}}{{\bar{\gamma }}_{R_{2}}}}+e^{\frac{-\gamma_{0}}{{\bar{\gamma }}_{R_{2}}}}erf\left(\sqrt{b\gamma_{0}}\right)-\sqrt{\frac{{\bar{\gamma }}_{R_{2}}b}{1+{\bar{\gamma }}_{R_{2}}b}}erf\left(\sqrt{\gamma_{0}\left(b+1/{\bar{\gamma }}_{R_{2}}\right)}\right)\right)}{\left({\bar{\gamma }}_{SD}-{\bar{\gamma }}_{R_{2}}\right)\left({\bar{\gamma }}_{R_{2}}-{\bar{\gamma }}_{R_{3}}\right)}+\right.\\ & & \left.\frac{{\bar{\gamma }}_{R_{1}}{\bar{\gamma }}_{R_{2}}^{2}\left(1-e^{\frac{-\gamma_{0}}{{\bar{\gamma }}_{R_{2}}}}+e^{\frac{-\gamma_{0}}{{\bar{\gamma }}_{R_{2}}}}erf\left(\sqrt{b\gamma_{0}}\right)-\sqrt{\frac{{\bar{\gamma }}_{R_{2}}b}{1+{\bar{\gamma }}_{R_{2}}b}}erf\left(\sqrt{\gamma_{0}\left(b+1/{\bar{\gamma }}_{R_{2}}\right)}\right)\right)}{\left({\bar{\gamma }}_{R_{1}}-{\bar{\gamma }}_{R_{2}}\right)\left({\bar{\gamma }}_{R_{2}}-{\bar{\gamma }}_{R_{3}}\right)}-\right.\\ & & \left.\frac{{\bar{\gamma }}_{SD}^{2}{\bar{\gamma }}_{R_{3}}\left(1-e^{\frac{-\gamma_{0}}{{\bar{\gamma }}_{R_{3}}}}+e^{\frac{-\gamma_{0}}{{\bar{\gamma }}_{R_{3}}}}erf\left(\sqrt{b\gamma_{0}}\right)-\sqrt{\frac{{\bar{\gamma }}_{R_{3}}b}{1+{\bar{\gamma }}_{R_{3}}b}}erf\left(\sqrt{\gamma_{0}\left(b+1/{\bar{\gamma }}_{R_{3}}\right)}\right)\right)}{\left({\bar{\gamma }}_{SD}-{\bar{\gamma }}_{R_{2}}\right)\left({\bar{\gamma }}_{SD}-{\bar{\gamma }}_{R_{3}}\right)}+\right.\\ & & \left.\frac{{\bar{\gamma }}_{R_{1}}^{2}{\bar{\gamma }}_{R_{3}}\left(1-e^{\frac{-\gamma_{0}}{{\bar{\gamma }}_{R_{3}}}}+e^{\frac{-\gamma_{0}}{{\bar{\gamma }}_{R_{3}}}}erf\left(\sqrt{b\gamma_{0}}\right)-\sqrt{\frac{{\bar{\gamma }}_{R_{3}}b}{1+{\bar{\gamma }}_{R_{3}}b}}erf\left(\sqrt{\gamma_{0}\left(b+1/{\bar{\gamma }}_{R_{3}}\right)}\right)\right)}{\left({\bar{\gamma }}_{R_{1}}-{\bar{\gamma }}_{R_{2}}\right)\left({\bar{\gamma }}_{R_{1}}-{\bar{\gamma }}_{R_{3}}\right)}+\right.\\ & & \left.\frac{{\bar{\gamma }}_{SD}{\bar{\gamma }}_{R_{2}}{\bar{\gamma }}_{R_{3}}\left(1-e^{\frac{-\gamma_{0}}{{\bar{\gamma }}_{R_{3}}}}+e^{\frac{-\gamma_{0}}{{\bar{\gamma }}_{R_{3}}}}erf\left(\sqrt{b\gamma_{0}}\right)-\sqrt{\frac{{\bar{\gamma }}_{R_{3}}b}{1+{\bar{\gamma }}_{R_{3}}b}}erf\left(\sqrt{\gamma_{0}\left(b+1/{\bar{\gamma }}_{R_{3}}\right)}\right)\right)}{\left({\bar{\gamma }}_{SD}-{\bar{\gamma }}_{R_{2}}\right)\left({\bar{\gamma }}_{R_{2}}-{\bar{\gamma }}_{R_{3}}\right)}-\right.\\ & & \left.\frac{{\bar{\gamma }}_{R_{1}}{\bar{\gamma }}_{R_{2}}{\bar{\gamma }}_{R_{3}}\left(1-e^{\frac{-\gamma_{0}}{{\bar{\gamma }}_{R_{3}}}}+e^{\frac{-\gamma_{0}}{{\bar{\gamma }}_{R_{3}}}}erf\left(\sqrt{b\gamma_{0}}\right)-\sqrt{\frac{{\bar{\gamma }}_{R_{3}}b}{1+{\bar{\gamma }}_{R_{3}}b}}erf\left(\sqrt{\gamma_{0}\left(b+1/{\bar{\gamma }}_{R_{3}}\right)}\right)\right)}{\left({\bar{\gamma }}_{R_{1}}-{\bar{\gamma }}_{R_{2}}\right)\left({\bar{\gamma }}_{R_{2}}-{\bar{\gamma }}_{R_{3}}\right)}\right] \end{array}$$

By substituting Equations (21), (24) and (27) or (30) or (38) in Equation (20), we get closed-form expression for error probability of incremental relaying with cooperative retransmissions. Note that *z* is an auxiliary variable which is representing $\gamma_{d}$, who is the sum of $\gamma_{SD}$ and $\gamma_{R_{i}}$.

**Figure 4:**
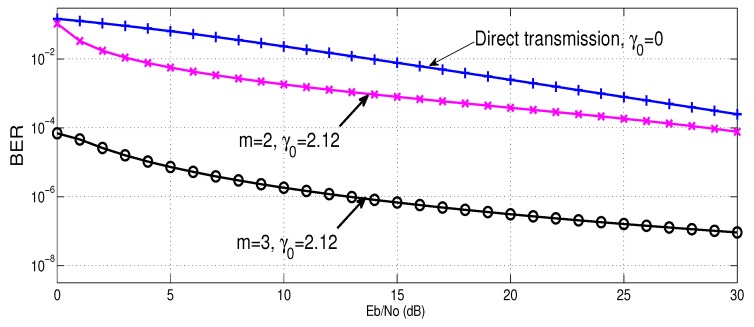
BER comparison of incremental relaying with cooperative retransmissions, using m = 2 and m = 3, with direct transmission.

Figure 4 shows the BER performance using incremental relaying cooperative diversity with cooperative retransmissions. The performance is meant for AF scheme using $\gamma_{0}=2.12$. In this plot, error probability for direct transmission, $\gamma_{0}$, and re-transmissions involving 2 and 3 relays are plotted against the SNR of the transmitted signal (Eb/No). Results demonstrate that because of diversity gain, the cooperation improves the BER performance in comparison with the direct transmission.

The results also show that in low SNR, system benefits from the diversity gain, however, at high SNR, both m = 2 and m = 3 tend to get parallel with the direct transmission. This is because, at high SNR destination rarely need assistance from relay to perform retransmission.

### 3.5. Proposed Scheme 1: ACE

**Figure 5:**
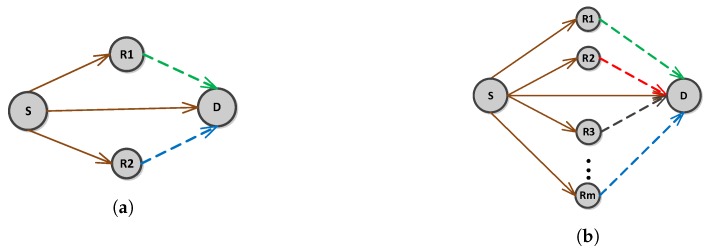
System model. (**a**) ACE; (**b**) E-ACE.

ACE is an adaptive cooperative retransmission routing protocol based on incremental relaying cooperative diversity. Figure 5a shows proposed system model of ACE for single S-D pair. It consists of S, D and two relays R1 and R2. S broadcasts data to D, R1 and R2 in the first phase. Retransmission from R1 and R2 is performed one by one in the second phase if and only if the destination receives erroneous signal at D. The proposed scheme works in time slots. A Time slot is a duration in which all alive nodes transmit their data to the Sink. It is assumed for the proposed protocol that alive nodes always have data to send. BS/Sink keeps the record of all the alive and dead nodes. It is the responsibility of BS to broadcast the Time Division Multiple Access (TDMA) schedule telling the nodes when it can transmit. Nodes send data packet during their allocated transmission time. When all the node finish data transmission, next time slot is initiated by the BS. Each slot consists of three phases given as:

1. Information exchange phase
2. Path establishment phase
3. Data transmission phase

#### 3.5.1. Information Exchange Phase

ACE is localization free routing protocol. Nodes are equipped with inexpensive depth sensors. Each node broadcasts its depth and residual energy information to all nodes in its transmission range via HELLO packet. Neighbour nodes are identified on the basis of depth information. A neighbor of node “x” is one whose depth difference from x lies within the transmission range of x. This process is repeated for all the nodes and information regarding local neighbours is stored in each node’s database.

Information exchange phase is repeated in the beginning of each time slot and neighbors and residual energy information is updated. This is because, once nodes start to die, neighbors and residual energy information may change and needs to be updated for the path establishment phase.

#### 3.5.2. Path Establishment Phase

Once a node knows its neighbours, a multi-hop path is established from source to sink. Path establishment phase has two main objectives; (i) identification of the next destination and (ii) identification of relays that act as cooperative nodes for data retransmission. Source node identifies its neighbours with the help of depth information of other nodes. Nodes that have depth lower than that of source node are included in the Forwarding Neighbour (FN) list and are called FNs which are potential candidates for next hop destination. Neighbours with depth greater than source node are neglected. EEDBR algorithm is followed to select the master node from FN list for the next hop destination. In this process, the node of lesser depth and highest residual energy is selected as master node.

Relay nodes are identified after the selection of master node and are identified among the nodes that lie in the cooperative region as shown in Figure 2. Nodes that are deployed in cooperative region are known as cooperative nodes. In ACE, only two nodes among all cooperative nodes act as relays. Selection criteria for R1 and R2 is based on highest residual energy and minimum depth. Once a node identifies the master node and cooperative nodes, it broadcasts this information to its neighbors. Neighbors on receiving this information know, who the master node is and who the cooperative nodes are. It is assumed that control messages are not lost during transmission. The process of choosing master node and retransmission nodes continues till sink is approached.

#### 3.5.3. Data Transmission Phase

**Figure 6:**
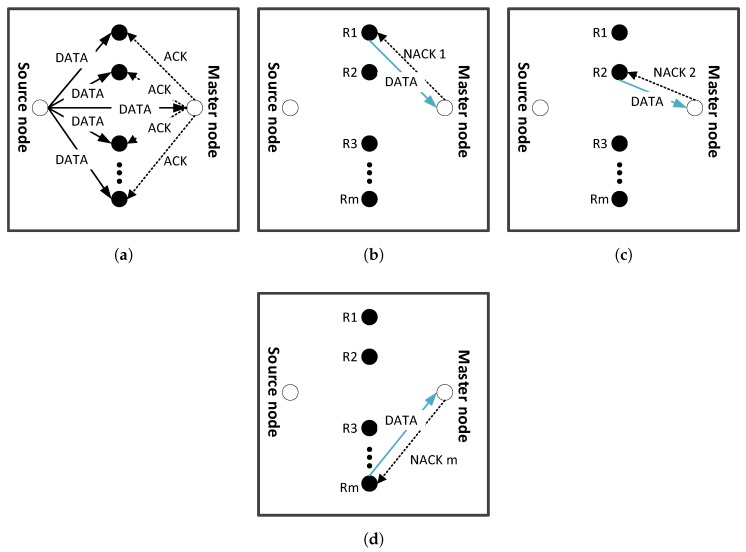
Data transmission from source to master node: (**a**) packet is accepted by master node and acknowledgement (ACK) is sent to retransmission nodes indicating that no retransmission is required; (**b**) packet is rejected by master node and asking for first retransmission from R1; (**c**) packet is again rejected and asking for second retransmission from R2; and (**d**) *m*th relay node performing data retransmission.

In this phase, data is transmitted from source to sink through the path which is established in path establishment phase. Source node broadcasts its data to master and cooperative nodes. Data on its way from source to destination suffers fading due to multi-path propagation and noise in the water. These factors introduce high BER in the signal. In ACE, data received at the master node in direct transmission is compared with the original data sent by the source node and BER is calculated. If BER is less than or equal to maximum allowable BER, *E*, then data packet is accepted. Upon acceptance, the master node sends acknowledgement (ACK) to retransmission nodes as shown in Figure 6a. Soon after, retransmission nodes discard the data.

If BER is greater than *E*, Negative ACK (NACK), is sent by master node to retransmission nodes. Figure 6b shows that, for the first retransmission, NACK1 is selectively sent to first retransmission node, R1. In response to NACK1, data is amplified and forwarded to master node by R1. This amplified data may also suffer fading and noise. Therefore, to achieve acceptable BER at master node, direct signal from source to master node and relayed signal from source to relay to master node are combined using MRC. At the master node, BER is calculated and compared with the predefined threshold, *E*. If it is less than or equal to *E*, data packet is accepted and ACK signal is sent to retransmission nodes. Retransmission nodes discard the data on reception of ACK signal. However, if BER is greater than *E*, NACK2 is sent to another retransmission node which amplifies and forwards the data to master node as shown in Figure 6c. Selection of different retransmission nodes leads to balanced energy consumption. A given node quickly depletes its energy if it is engaged in multiple retransmissions. At the master node, the direct, and two relayed signals are combined using MRC. If the calculated BER is less than or equal to *E*, data packet is accepted else dropped. Incase of ACK from the master node, the retransmission nodes discard their data. It is assumed that signaling information; ACK/NACK is not lost during transmission.

### 3.6. Proposed Scheme 2: E-ACE

In poor underwater environment (noise, water bubbles, fading, etc), high BER is introduced that make two retransmissions insufficient. Therefore, to increase reliability and throughput efficiency of the network, E-ACE is proposed. Unlike ACE, E-ACE allows *m* relays to perform cooperative retransmissions as shown in Figure 5b. Instead of using two cooperative nodes, as in ACE, for retransmissions, rest of the nodes, present in the cooperative region, are used. In this way energy balancing is achieved and node does not deplete its energy in performing multiple retransmissions. ACE is actually a special case of E-ACE with cooperative nodes limited to 2.

Rest of the E-ACE protocol operation is similar to that of ACE, i.e., information exchange phase, path establishment phase, and data transmission phase. It is worth mentioning in E-ACE that all the nodes present in cooperative region are candidates for retransmitting the data. The nodes in cooperative region may vary for each S-D pair. More *m* results in increased retransmissions, thereby, reducing outage probability and vice versa.

#### 3.6.1. Information Exchange Phase

This phase is replica of ACE information exchange phase.

#### 3.6.2. Path Establishment Phase

This phase is similar to ACE’s path establishment phase.

#### 3.6.3. Data Transmission Phase

**Figure 7:**
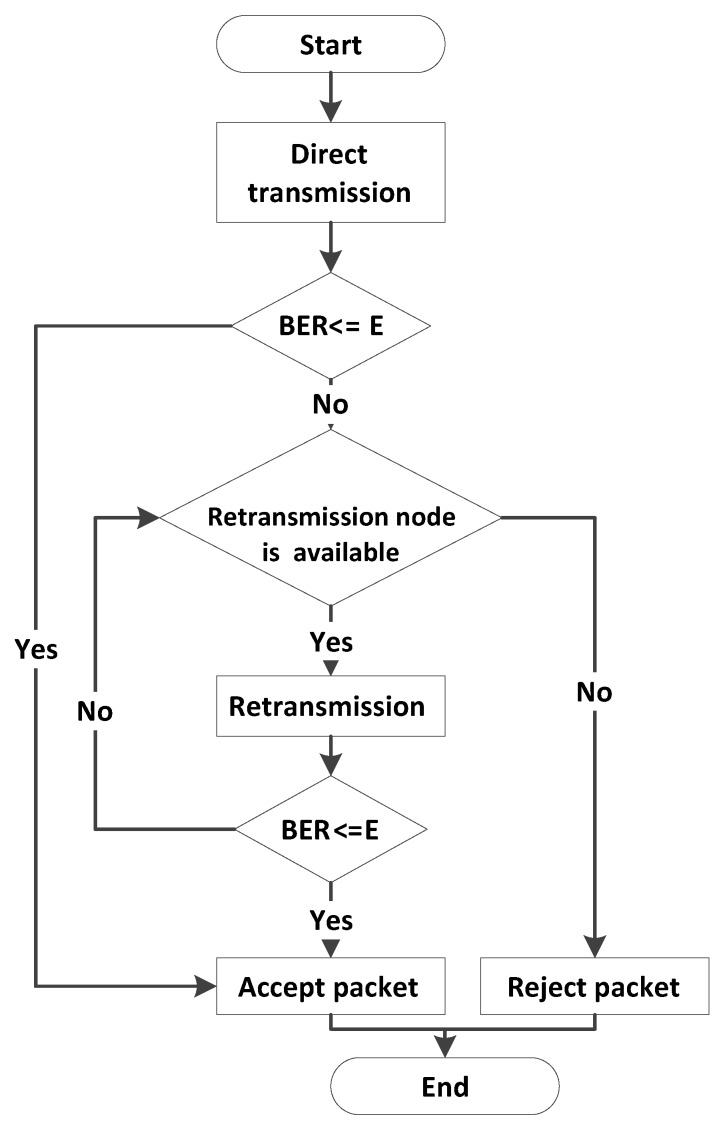
Data transmission flow chart for Enhanced-Adaptive Cooperation in Energy (E-ACE).

Till 2nd retransmission, this phase is similar to ACE’s data transmission phase. However, if BER is not acceptable after retransmission from R2, R3 does data retransmission and the process continues till all the retransmission nodes are expired or acceptable BER is achieved as shown in Figure 6d. Figure 7 presents data transmission flow chart of a single packet for E-ACE.

### 3.7. Energy Consumption Analysis

In this section, we evaluate the energy consumption of ACE and E-ACE.

The transmission energy ET, for a message of size *L* bits through a channel with bandwidth, *B* kbps is given as [31]:

(39) $$ET=P_{t}\times \frac{L}{B}$$

Similarly, the receiving energy, ER, is defined as:

(40) $$ER=P_{r}\times \frac{L}{B}$$

where $P_{t}$ and $P_{r}$ are transmit and receive power respectively. Let *N* be the total number of nodes in the network and all nodes always have data to transmit. The total transmission energy $ET_{TX}$ with m relays involved in data transmission becomes,

(41) $$ET_{TX}=\left(m+1\right)\times ET$$

Here the term (m+1), *m* indicates the number of relays involved in retransmission and 1 indicate the transmission from source node. In case of ACE, m=1,2. If no retransmission is performed, m=0. Similarly the total reception energy, $ER_{RX}$ is derived as:

(42) $$ER_{RX}=\left(m+1\right)\times ER$$

In reception energy, 1 indicates the reception at master node.

The transmission from source to sink takes place in a multi-hop manner. The number of hops can be different for different nodes depending on node’s location. Let $h_{i}$ be the number of hops for data packet of node $N_{i}$. The energy required for data from node $N_{i}$ travelling through $h_{i}$ hops is given as:

(43) $$ET_{TXi}=N_{i}\times h_{i}\times \left(m+1\right)\times ET$$

where, i=1:N. The total transmission energy of the network becomes:

(44) $$ET_{TX}=\sum_{i=1}^{N}N_{i}\times h_{i}\times \left(m+1\right)\times ET$$

Similarly the total reception energy, $ER_{RX}$ is given as:

(45) $$ET_{RX}=\sum_{i=1}^{N}N_{i}\times h_{i}\times \left(m+1\right)\times ER$$

In ACE and E-ACE, besides data transmission, we have energy spent by control messages. Let $E_{CTRL}$ be the total energy used in control messages. The total energy consumption for ACE and E-ACE is,

(46) $$E=ET_{TX}+ER_{RX}+E_{CTRL}$$

where, $E_{CTRL}$, with length of control message LC, is defined as:

(47) $$E_{CTRL}=\left(\sum_{i=1}^{N}N_{i}\times h_{i}\right)P_{t}\times \frac{LC}{B}+\left(\sum_{i=1}^{N}\left(N_{i}+m\right)\times h_{i}\right)P_{r}\times \frac{LC}{B}$$

## 4. Simulation Results and Discussions

In this section, the performance of the proposed protocols ACE and E-ACE are compared with other proposed protocols referred as EEDBR [3] and Cooperative ARQ (CARQ) [11]. CARQ is is an ARQ based scheme in which cooperative nodes are used to provide alternative paths for a specific source destination link. Relay nodes are selected based on the smallest distance from the destination. When the destination node receives an erroneous packet, it asks for retransmission from a cooperative node, which is selected in a closest-one-first manner from the nodes in the cooperative region. EEDBR is another routing protocol that takes depth and residual energy information in selection of next hop. It is a non cooperative routing protocol and relies on single link for data transmission.z

In simulations, 250 nodes are randomly deployed in an area of 500 m × 500 m. The network is homogenous and each node has initial energy of 30 J and a fixed transmission range of 100 m. The values of energy consumption are 2 W for transmission, 0.1 W for reception and 10 mW for idle sensing. Maximum allowable BER is set to 0.49 and *m* = 3. Channel bandwidth is 30 kbps. The size of data packet is 1000 bits and that of control packet is 48 bits. We assume that the nodes are anchored with wires in seawater such that the nodes can slightly move horizontally and the horizontal movement is small enough to be neglected. Four unconstrained sinks (in terms of energy) are deployed on the surface of water at an equal distance of 100 m. Underwater nodes are equipped with acoustic modems and sinks are equipped with both acoustic and radio modems. It is worth mentioning here that only for the sake of fair comparison, the simulation parameters are chosen according the existing works used for comparison.

### 4.1. Network Performance Parameters—Definitions

Subject to validation of the proposed work, we conduct simulations in terms of the following metrics.

1. *Network lifetime:* It is the time duration from the start of the network till the death of the last node.
2. *Total energy consumption:* It is the total energy consumed by all the nodes during transmission, reception, and idle time. It is measured in Joules.
3. *Throughput:* It is defined as the total number of successfully received packets at the sink and is measured in packets/time slot.
4. *Packet drop:* It is the number of packets that are not successfully received at the sink.
5. *Packet acceptance ratio:* It is the ratio of packets received at sink to the total number of packets sent towards sink.

### 4.2. Network Performance Parameters—Discussion

#### 4.2.1. Network Lifetime

**Figure 8:**
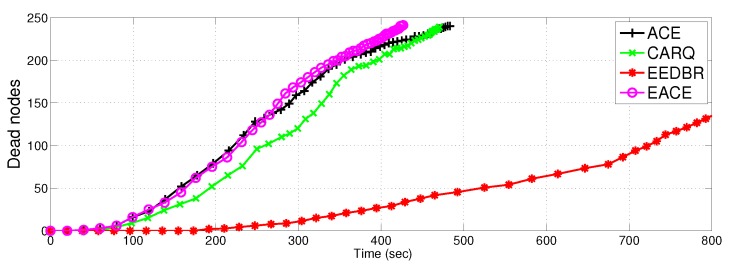
Network lifetime.

Figure 8 shows network lifetime comparison of the proposed protocols with EEDBR and CARQ. Network lifetime of ACE, E-ACE and CARQ is less than EEDBR because they consume more energy in retransmissions in case of erroneous data reception. Difference in lifetime of EEDBR and ACE is greater, whereas, the difference in lifetime of ACE, E-ACE an CARQ is small. This is due to the fact that ACE and CARQ allows only two relays and hence, maximum two retransmissions can be done, whereas, E-ACE allows *m* relays to retransmit data packet. However, most of the time two retransmissions are enough to achieve acceptable BER at destination and in some cases (low SNR region, obstacle, etc.), there is requirement for three or more retransmissions. This also validates our assumption of limiting *m* to 3.

Although, both CARQ and ACE are using two retransmission nodes, however, lifetime of ACE is less than CARQ because, ACE has more throughput.The reason behind is that ACE use MRC technique to combine data from direct transmission and relayed transmissions. MRC significantly reduces the BER introduced during individual transmissions. Signal combining technique is not incorporated in CARQ, therefore, data does not achieve acceptable BER. Hence packet is forcefully dropped at the same hop even after 2 retransmissions and protocol continues with the next node packet.

#### 4.2.2. Total Energy Consumption

**Figure 9:**
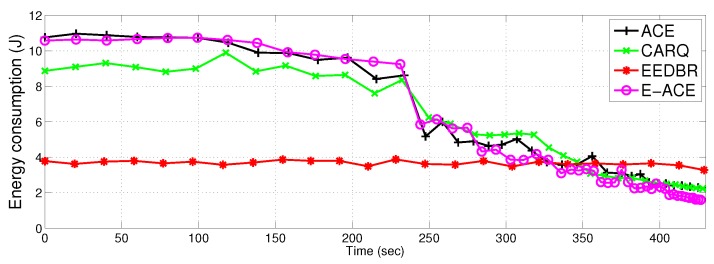
Total energy consumption of the network.

**Figure 10:**
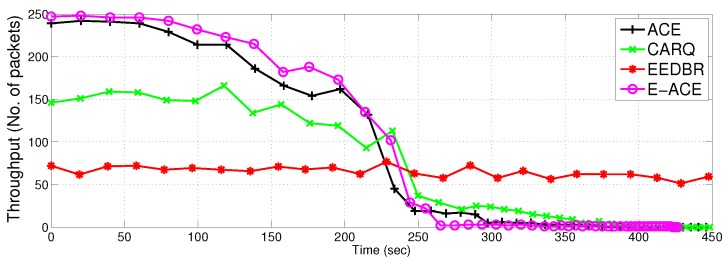
Throughput.

**Figure 11:**
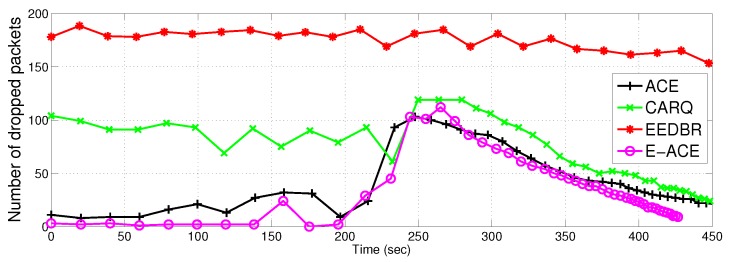
Packets dropped.

Figure 9 shows total energy consumption of the proposed protocols in comparison to EEDBR and CARQ. Energy consumption of E-ACE, ACE and CARQ is greater than EEDBR due to more allowed retransmissions. ACE and E-ACE show same energy consumption with almost same throughput (refer Figure 10). CARQ and EEDBR show less energy consumption because they have low throughput and high packet drop (refer Figure 11). Less throughput contributes to low energy consumption because less packets are transmitted.

In later time slots, energy consumption of the four protocols is almost same because ACE and E-ACE have very few alive nodes to send data to sink. EEDBR has 238 nodes alive at 300 s, whereas, ACE and E-ACE have not more than 100 alive nodes.

Figure 8 and Figure 9 show the drawback of E-ACE. These figures also show the tradeoff between energy consumption and reliability. More number of retransmission causes surplus energy consumption, thereby, reducing the network lifetime.

#### 4.2.3. Throughput

Figure 10 shows the performance of ACE and E-ACE in terms of throughput. E-ACE has 69% more throughput as compared to CARQ and ACE has 63% more throughput as compared to CARQ. Difference in throughput of E-ACE and ACE is less because third retransmission is rarely invoked. Most of the time, two retransmissions are enough to achieve acceptable BER. CARQ and EEDBR shows less throughput as compared to ACE and E-ACE, because network conditions (noise, fading, etc.) may not be suitable to achieve acceptable BER even after retransmissions in CARQ. CARQ does not combine the source signal with the relayed signal to reduce BER. When direct transmission is not successful, it discards the erroneous data and rely only on the retransmitted data packet from relay.

**Figure 12:**
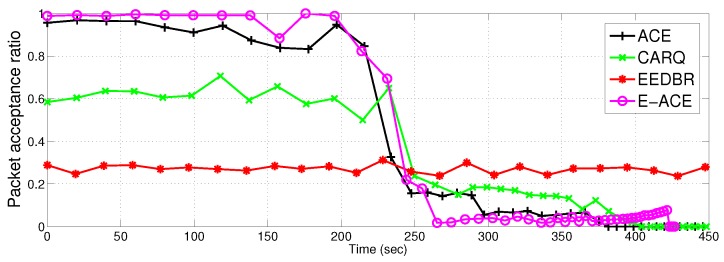
Packet acceptance ratio.

Table 2 shows throughput vs. dead nodes comparison of the four protocols. It shows that initially E-ACE has more throughput than ACE, however, this difference is very small. The performance of ACE and E-ACE is better than EEDBR till the death of 130 nodes. It is observed that throughput can be increased by using more cooperative nodes. However, with more retransmissions, more energy is consumed. After 230 s, ACE, E-ACE and CARQ have less throughput as compared to EEDBR because they have less number of alive nodes available to send data to sink, whereas, EEDBR has more alive nodes. More alive nodes can send more data to sink and increase throughput. Since the load on nodes is equally distributed, therefore, in sparse network, nodes may not find relays or next destination to send data to sink and die due to idle sensing. This is the main reason of low throughput and low packet acceptance ratio as shown in later time slots of Figure 12.

#### 4.2.4. Packet Drop

Figure 11 compares E-ACE, ACE with CARQ and EEDBR in terms of packet drop. It is actually the difference between total number of packets sent to sink and the total number of packets successfully received at sink. In simulations, packet is considered dropped when BER at destination is greater than the threshold after double or triple retransmissions. Packet is also considered as dropped when there is no neighbour to cooperate. As each node sends single packet per time slot and there is no data aggregation at relay nodes, therefore, maximum number of sent packets to sink are equal to total number of nodes.

In ACE, packet drop rate is negligible and close to E-ACE. Whereas, EEDBR has packet drop rate close to 70%. Figure 11 shows performance improvement of E-ACE as compared to EEDBR and CARQ. EEDBR relies on single link for data transmission and no cooperative mechanism is involved. Therefore, it has maximum packet drop count. EEDBR shows increase in packet drop rate in the later course of simulations due to availability of fewer alive nodes for data transportation to sink. CARQ shows more packet drop despite of using retransmission mechanism using two nodes because of non incorporation of data combining technique at the destination. Also, the relay selection mechanism in CARQ is dependant on distance only, therefore, energy balancing is not achieved and cooperative node may nodes die out who was responsible for retransmission.

#### 4.2.5. Packet Acceptance Ratio

Figure 12 demonstrates another parameter, i.e., packet acceptance ratio. E-ACE outperforms EEDBR with packet acceptance ratio close to 98%. Whereas, EEDBR has the lowest packet acceptance ratio (approximately 28%). This means that less than half of the packets are successfully delivered to sink and rest of the packets are dropped. ACE shows acceptance ratio of 95% and CARQ shows acceptance ratio of 58%. After 230 s, there is a drop off in acceptance ratio of ACE, E-ACE and CARQ. This drop is due to two reasons; (1) less relays are available to provide cooperative retransmissions and (2) due to fading consideration, the channel conditions are not suitable to achieve BER more than the threshold even after two retransmissions.

More acceptance ratio means more reliable network. In later time slots, EEDBR has more packet acceptance ratio because it has more alive nodes to forward data to sink. Thus, the retransmission mechanism clearly enhances network performance in terms of reliability.

### 4.3. Performance Trade-offs

In this section, trade-offs (the cost at which the performance metrics are achieved) of the proposed protocols are discussed (refer to Table 3).

ACE achieves more throughput by compromising on the network lifetime. Figure 8 and Figure 9 demonstrate the decrease in lifetime and increase in energy consumption of the network respectively. In case of direct transmission failure, retransmission from the relay nodes causes increased energy consumption thereby reducing network lifetime. It can be clearly seen that when throughput is maximum in the initial seconds (see Figure 10), the energy consumption is more than double of EEDBR protocol. This is because transmission energy is used once or twice more for retransmission of data. After 230 s, when more than 100 nodes are dead in ACE (Figure 8), throughput is decreased, hence energy consumption is also decreased. Later on, energy consumption of ACE is almost similar to EEDBR and CARQ with throughput less than EEDBR. Decrease in throughput is due to less number of alive nodes to carry data to sink.

E-ACE achieves throughput and reliability at the cost of reduced network lifetime. The reduction in network lifetime is due to increased energy consumption; retransmissions in case of direct transmission failure. Before 230 s (Figure 9), Relay transmissions are activated whenever direct transmission fails to achieve an acceptable BER, that is why, energy consumption of E-ACE is almost twice as compared to EEDBR (refer Figure 10). Later on, EEDBR and CARQ outperforms E-ACE (refer Figure 12) because they have more alive nodes to carry data to sink. Thus, we can say that E-ACE has less throughput and packet acceptance ratio than EEDBR, however, at the cost of more energy consumption. Reason behind this observation is that nodes decay rate of EEDBR is very slow as compared to E-ACE. Therefore, energy required by all the nodes for single transmission in EEDBR is almost equal to energy required for cooperative retransmissions in E-ACE.

In both protocols, throughput is increased at the price of increased time delay. Cooperative diversity is implemented in time division channel. Source node broadcasts to destination and relay nodes in first phase. In case of direct transmission failure, first retransmission is performed by the retransmission node in the second phase, thus adding more delay. If it is again not acceptable, next retransmission is performed in the third phase, adding another delay, and so on. Therefore, more number of retransmissions enhance reliable reception, however, at the cost of surplus delay.

## 5. Conclusions and Future Work

In this paper, incremental relaying with cooperative retransmission protocols; ACE and E-ACE, have been proposed for UWSNs along with outage and error probability performance analysis. Closed-form expressions for outage and error probability are determined and expression for the number of available relays is also derived. Results show that incremental relaying with triple retransmissions shows very less outage probability and so is BER as compared to regular incremental relaying cooperative diversity network having single retransmission mechanism. The proposed model and protocols are validated via simulations. We evaluated the proposed schemes by comparing them with another cooperative protocol called CARQ and non cooperative routing protocol referred as EEDBR. ACE and E-ACE substantially show better relative performance in terms of reliability and throughput efficiency. This improvement is likely due to the cooperative diversity benefits and the retransmission mechanism. However, this reliability is achieved at the cost of increased energy consumption which leads to decreased network lifetime. Future works include derivation of expression capacity of incremental relaying with *m* retransmissions. In addition, expressions for outage, BER and capacity using DF incremental relaying cooperative retransmissions will also be derived.

This research work assumed non-LOS communication between static nodes, so, in future, we will extend our work for LOS communication between mobile nodes.

## Figures and Tables

**Table 1:** Comparison of the Related Work.

Ref.		Issues Addressed	Technique	Relaying Technique	Flaws/ Deficiencies	Achievements	Cost
[12]	WSNs	packet collision minimization, Collision caused by source and relay	Branch and bound, Mixed integer non-linear programming, CSMA-CA	Incremental decode and forward	only a single cost function is used	Minimum collision probability, minimized total transmission power	Energy consumption
[13]	WSNs	Dependence of the ergodic capacity, outage probability	Pareto efficient scheme, channel state information	Decode and forward	Only single relay is used	Increase in channel capacity	Overhead
[14]	UWSNs	Overloading of acoustic channel, Hidden terminal problem	Greedy algorithm, Opportunistic routing	Decode and forward	Longer Delay, Additional energy consumption	Fraction of void node is decreased, Reduced redundant packets	End-to-end delay, Energy consumption
[15]	WSNs	Inefficient utilization of channel	Incremental relaying cooperative diversity	Amplify and forward	No end-to-end delay analysis	Reduced bit error rate, High throughput	Outage increases
[16]	WSNs	Energy consumption for acquiring CSI	Relay cooperative beam forming, Channel state information	Decode and forward	Tradeoff between energy consumption and data transmission	Energy efficiency	Overhead of channel state information
[17]	WSNs	Energy consumption per data packet, Maximize the network lifetime	RTS/CTS signaling	Decode and forward	Overhead of RTS/CTS signaling	Energy efficiency, Prolonged network lifetime	Delay
[18]	WSNs	Relaying selection parameter	Onion layered cooperation, Information theory	Decode and forward, Amplify and forward	Increase overhead and extensive computation	New relaying mechanism	High energy consumption
[19]	WSNs	Overall outage probability, Average symbol error rate	Binary phase shift keying, Monte carlos simulations	Amplify and forward		Optimal values for OOP and ASEP	
[20]	WSNs	Relay selection technique, Optimal power for source and relay nodes	Channel state information, Orthogonal channel model	Decode and forward	To decrease SER, Number of relays must be increased	New SER method, Throughput	Network lifetime
[21]	WSNs	Single hop cooperation, Relay node assignment	Branch and cut, Orthogonal channel model, Linear integer programming	Amplify and forward	Interference between two or more sessions	Throughput	Delay, Network lifetime
[22]	UWSNs	Good put and end-to-end latency	Opportunistic routing	Direct method	SNR,PER,BER not taken as a performance metrics	Less end-to-end delay	Energy consumption
[23]	WSNs	Packet collision minimization	Cooperative routing, Bellman-ford equation	incremental adaptive decode and forward	No optimal selection of relays	Optimal power allocation	Energy consumption
[24]	WSNs	Optimal transmission policy, Low computational complexity	Multi parametric programming, QPSK	Decode and forward	Less power efficient for large scale networks	Low BER, Low transmission power	Not suitable for higher throughput applications
[25]	WSNs	Total energy consumption in clustered network	Space time block code, BPSK	Decode and forward	Short network lifetime	Minimizing overall energy consumption	Packet error rate
[26]	WSNs	Saving energy	Reducing total number of transmissions, Shortening delay	Node cooperation, Cross layer	Direct relay system	No residual energy check procedure, Not suitable for sparse network	Energy saving, Ack exchange overhead

**Table 2:** Throughput vs. dead nodes.

Serial. No.	Protocol Name	Dead Nodes					
		0	50	100	150	200	250
1	E-ACE	247	182	102	26	3	0
2	ACE	239	166	46	15	3	0
3	EEDBR	72	59	17	2	1	0
4	CARQ	146	119	29	15	1	0

**Table 3:** Performance trade-offs for ACE and E-ACE.

Protocol	Achieved Performance Parameter	Cost Paid
E-ACE	Highest throughput, least packet drop,	Highest delay, short network lifetime,
	highest reliability.	high energy consumption, high complexity.
ACE	High throughput, less packet drop,	High delay, short network lifetime,
	high packet acceptance ratio, high reliability.	high energy consumption, complexity.
EEDBR	Low throughput, low reliability,	Low delay, long network lifetime,
	high packet drop.	less energy consumption, low complexity.
CARQ	Medium throughput, medium reliability,	Low delay, short network lifetime,
	medium packet drop.	high energy consumption, medium complexity.

**Figure A1:**
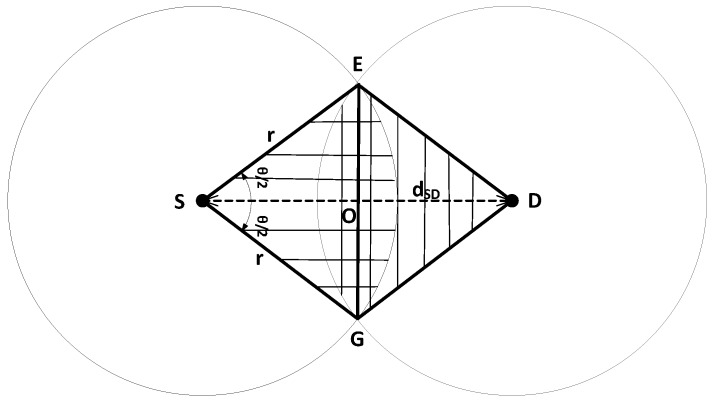
Area of overlapping region.

## Appendix A. Area of Overlapping Region

Figure A1 shows the overlapping area of same transmission ranges of two sensors. Transmission range is denoted by *r*. Therefore, the area of overlapping region is found by subtracting area of triangle SEG from area of sector [32].

Area of sector is given by:

(A1) $$AS=\frac{\theta }{2}r^{2}$$

where *θ* is the angle of sector in radians. In order to find *θ*, we know that triangle SEG is an isosceles triangle with height $d_{SD}/2$. Using half angle identity:

(A2) $$\begin{array}{ccc} \cos(\frac{\theta }{2}) & = & \frac{d_{SD}/2}{r}=\frac{d_{SD}}{2r} \end{array}$$

solving for *θ*, we get:

(A3) $$\theta =2\arccos(\frac{d_{SD}}{2r})$$

Putting Equation (A3) in Equation (A1), we get:

(A4) $$\begin{array}{ccc} AS & = & \frac{2\arccos(\frac{d_{SD}}{2r})}{2}r^{2}=r^{2}\arccos\left(\frac{d_{SD}}{2r}\right) \end{array}$$

Now area of triangle SEG is given as:

(A5) $$AT=\frac{1}{2}\;base\times height.$$

Using Pythagorean theorem to find $\bar{OE}$ of the right angle triangle SOE.

(A6) $$\bar{OE}=\sqrt{r^{2}-\frac{d_{SD}^{2}}{4}}$$

Therefore, base of the triangle SEG is $2\times \bar{OE}$.

(A7) $$base=\sqrt{4r^{2}-{d_{SD}}^{2}}$$

Area of the triangle SEG is given as:

(A8) $$\begin{array}{ccc} AT & = & \frac{1}{2}\sqrt{4r^{2}-d_{SD}^{2}}\times \frac{d_{SD}}{2}\\ & = & \frac{d_{SD}}{4}\sqrt{4r^{2}-{d_{SD}}^{2}} \end{array}$$

Area of the overlapping region is 2×(AS-AT).

(A9) $$\begin{array}{ccc} A & = & 2\times \left[r^{2}\arccos\left(\frac{d_{SD}}{2r}\right)-\frac{d_{SD}}{4}\sqrt{4r^{2}-{d_{SD}}^{2}}\right]\\ A & = & 2r^{2}\arccos\left(\frac{d_{SD}}{2r}\right)-\frac{d_{SD}}{2}\sqrt{4r^{2}-d_{SD}^{2}} \end{array}$$

## Appendix B. Derivation of *f_γd_*

*γ* follows exponential distribution with mean $\bar{\gamma }$. By definition of PDF:

(B1) $$f_{\gamma_{SD}}=\frac{1}{{\bar{\gamma }}_{SD}}exp\left(-\gamma_{SD}/{\bar{\gamma }}_{SD}\right)$$

(B2) $$f_{\gamma_{R_{i}}}=\frac{1}{{\bar{\gamma }}_{R_{i}}}exp\left(-\gamma_{R_{i}}/{\bar{\gamma }}_{R_{i}}\right),⋁\;i\;\epsilon \left\{1,2,3\right\}$$

$f_{\gamma_{d}}$ is calculated by the convolution of above equations. By parts convolution, $f_{\gamma_{1}}\left(x\right)$ is derived as [15]:

(B3) $$\begin{array}{ccc} f_{\gamma_{1}}\left(x\right) & = & \frac{1}{{\bar{\gamma }}_{SD}}exp\left(-x/{\bar{\gamma }}_{SD}\right)*\frac{1}{{\bar{\gamma }}_{R_{1}}}exp\left(-x/{\bar{\gamma }}_{R_{1}}\right)\\ & = & \frac{1}{{\bar{\gamma }}_{SD}{\bar{\gamma }}_{R_{1}}}\int_{0}^{z}exp\left(\frac{-{\bar{\gamma }}_{R_{1}}x-{\bar{\gamma }}_{SD}z+{\bar{\gamma }}_{SD}x}{{\bar{\gamma }}_{SD}{\bar{\gamma }}_{R_{1}}}\right)dx\\ & = & \frac{1}{{\bar{\gamma }}_{SD}-{\bar{\gamma }}_{R_{1}}}exp\left(-z/{\bar{\gamma }}_{SD}\right)-exp\left(-z/{\bar{\gamma }}_{R_{1}}\right) \end{array}$$

Similarly, convolution of $f_{\gamma_{1}}$ and $f_{\gamma_{R_{2}}}$ is calculated as:

(B4) $$\begin{array}{ccc} f_{\gamma_{2}}\left(x\right) & = & \frac{1}{{\bar{\gamma }}_{SD}-{\bar{\gamma }}_{R_{1}}}exp\left(-x/{\bar{\gamma }}_{SD}\right)-exp\left(-x/{\bar{\gamma }}_{R_{1}}\right)*\frac{1}{{\bar{\gamma }}_{R_{2}}}exp\left(-x/{\bar{\gamma }}_{R_{2}}\right)\\ & = & \frac{1}{{\bar{\gamma }}_{R_{2}}\left({\bar{\gamma }}_{SD}-{\bar{\gamma }}_{R_{1}}\right)}\int_{0}^{z}\left[\left[exp(\frac{-{\bar{\gamma }}_{R_{2}}x-{\bar{\gamma }}_{SD}z+{\bar{\gamma }}_{SD}x}{{\bar{\gamma }}_{R_{2}}{\bar{\gamma }}_{SD}})\right]-\left[exp(\frac{-{\bar{\gamma }}_{R_{2}}x-{\bar{\gamma }}_{R_{1}}z+{\bar{\gamma }}_{R_{1}}x}{{\bar{\gamma }}_{R_{1}}{\bar{\gamma }}_{R_{2}}})\right]\right]dx\\ & = & \frac{\left[\frac{{\bar{\gamma }}_{SD}}{{\bar{\gamma }}_{SD}-{\bar{\gamma }}_{R_{2}}}\left[exp\left(-z/{\bar{\gamma }}_{SD}\right)-exp\left(-z/{\bar{\gamma }}_{R_{2}}\right)\right]-\frac{{\bar{\gamma }}_{R_{1}}}{{\bar{\gamma }}_{R_{1}}-{\bar{\gamma }}_{R_{2}}}\left[exp\left(-z/{\bar{\gamma }}_{R_{1}}\right)-exp\left(-z/{\bar{\gamma }}_{R_{2}}\right)\right]\right]}{\left({\bar{\gamma }}_{SD}-{\bar{\gamma }}_{R_{1}}\right)} \end{array}$$

Third convolution of $f_{\gamma_{2}}$ with $f_{\gamma_{R_{3}}}$ is calculated as:

(B5) $$\begin{array}{ccc} f_{\gamma_{3}}\left(x\right) & = & \frac{\left[\frac{{\bar{\gamma }}_{SD}}{{\bar{\gamma }}_{SD}-{\bar{\gamma }}_{R_{2}}}\left[exp\left(-x/{\bar{\gamma }}_{SD}\right)-exp\left(-x/{\bar{\gamma }}_{R_{2}}\right)\right]-\frac{{\bar{\gamma }}_{R_{1}}}{{\bar{\gamma }}_{R_{1}}-{\bar{\gamma }}_{R_{2}}}\left[exp\left(-x/{\bar{\gamma }}_{R_{1}}\right)-exp\left(-x/{\bar{\gamma }}_{R_{2}}\right)\right]\right]}{\left({\bar{\gamma }}_{SD}-{\bar{\gamma }}_{R_{1}}\right)}\\ & & *\frac{1}{{\bar{\gamma }}_{R_{3}}}exp\left(-x/{\bar{\gamma }}_{R_{3}}\right)\\ & = & \frac{{\bar{\gamma }}_{SD}}{{\bar{\gamma }}_{R_{3}}\left({\bar{\gamma }}_{SD}-{\bar{\gamma }}_{R_{2}}\right)}\int_{0}^{z}\frac{\left[exp\left(\frac{-{\bar{\gamma }}_{R_{3}}x-{\bar{\gamma }}_{SD}z+{\bar{\gamma }}_{SD}x}{{\bar{\gamma }}_{SD}{\bar{\gamma }}_{R_{3}}}\right)-exp\left(\frac{-{\bar{\gamma }}_{R_{3}}x-{\bar{\gamma }}_{R_{2}}z+{\bar{\gamma }}_{R_{2}}x}{{\bar{\gamma }}_{R_{2}}{\bar{\gamma }}_{R_{3}}}\right)\right]dx}{{\bar{\gamma }}_{SD}-{\bar{\gamma }}_{R_{1}}}\\ & \; & -\frac{{\bar{\gamma }}_{R_{1}}}{{\bar{\gamma }}_{R_{3}}\left({\bar{\gamma }}_{R_{1}}-{\bar{\gamma }}_{R_{2}}\right)}\int_{0}^{z}\frac{\left[exp\left(\frac{-{\bar{\gamma }}_{R_{3}}x-{\bar{\gamma }}_{R_{1}}z+{\bar{\gamma }}_{R_{1}}x}{{\bar{\gamma }}_{R_{1}}{\bar{\gamma }}_{R_{3}}}\right)-exp\left(\frac{-{\bar{\gamma }}_{R_{3}}x-{\bar{\gamma }}_{R_{2}}z+{\bar{\gamma }}_{R_{2}}x}{{\bar{\gamma }}_{R_{2}}{\bar{\gamma }}_{R_{3}}}\right)\right]dx}{{\bar{\gamma }}_{SD}-{\bar{\gamma }}_{R_{1}}} \end{array}$$

Integrating and doing some necessary manipulations, PDF of $f_{\gamma_{d}}$ is given by:

(B6) $$\begin{array}{ccc} f_{\gamma_{d}} & = & \frac{{\bar{\gamma }}_{SD}^{2}\left[\exp\left(-z/{\bar{\gamma }}_{SD}\right)-\exp\left(-z/{\bar{\gamma }}_{R_{3}}\right)\right]}{\left({\bar{\gamma }}_{SD}-{\bar{\gamma }}_{R_{1}}\right)\left({\bar{\gamma }}_{SD}-{\bar{\gamma }}_{R_{2}}\right)\left({\bar{\gamma }}_{SD}-{\bar{\gamma }}_{R_{3}}\right)}-\\ & & \frac{{\bar{\gamma }}_{SD}{\bar{\gamma }}_{R_{2}}\left[\exp\left(-z/{\bar{\gamma }}_{R_{2}}\right)-\exp\left(-z/{\bar{\gamma }}_{R_{3}}\right)\right]}{\left({\bar{\gamma }}_{SD}-{\bar{\gamma }}_{R_{1}}\right)\left({\bar{\gamma }}_{SD}-{\bar{\gamma }}_{R_{2}}\right)\left({\bar{\gamma }}_{R_{2}}-{\bar{\gamma }}_{R_{3}}\right)}+\\ & & \frac{{\bar{\gamma }}_{R_{1}}\left[\upsilon +\tau \right]}{\left({\bar{\gamma }}_{SD}-{\bar{\gamma }}_{R_{1}}\right)\left({\bar{\gamma }}_{R_{1}}-{\bar{\gamma }}_{R_{2}}\right)} \end{array}$$

where:

(B7) $$\upsilon =\frac{{\bar{\gamma }}_{R_{2}}\left[\exp\left(-z/{\bar{\gamma }}_{R_{2}}\right)-\exp\left(-z/{\bar{\gamma }}_{R_{3}}\right)\right]}{{\bar{\gamma }}_{R_{2}}-{\bar{\gamma }}_{R_{3}}}$$

and

(B8) $$\tau =\frac{{\bar{\gamma }}_{R_{1}}\left[\exp\left(-z/{\bar{\gamma }}_{R_{1}}\right)-\exp\left(-z/{\bar{\gamma }}_{R_{3}}\right)\right]}{{\bar{\gamma }}_{R_{1}}-{\bar{\gamma }}_{R_{3}}}$$

## Appendix C. Derivation of *P_out_*

The general expression for outage probability derived in Section 3.3 Equation (11) is given as:

(C1) $$\begin{array}{ccc} P_{out} & = & Pr\left(\gamma_{SD}\leq \gamma_{0}\right)Pr\left(\gamma_{SR_{1}D}+\gamma_{SD}\leq \gamma_{0}|\gamma_{SD}\leq \gamma_{0}\right)\\ & & Pr(\gamma_{SR_{1}D}+\gamma_{SR_{2}D}+\gamma_{SD}\leq \gamma_{0}|\gamma_{SR_{1}D}+\gamma_{SD}\leq \gamma_{0})\cdots \\ & & Pr(\sum_{i=1}^{m}\gamma_{SR_{i}D}+\gamma_{SD}\leq \gamma_{0}|\sum_{i=1}^{m-1}\gamma_{SR_{i}D}+\gamma_{SD}\leq \gamma_{0}) \end{array}$$

By limiting *m* to 3, we get:

(C2) $$\begin{array}{ccc} P_{out} & = & Pr\left(\gamma_{SD}\leq \gamma_{0}\right)Pr\left(\gamma_{SR_{1}D}+\gamma_{SD}\leq \gamma_{0}|\gamma_{SD}\leq \gamma_{0}\right)\\ & & Pr(\gamma_{SR_{1}D}+\gamma_{SR_{2}D}+\gamma_{SD}\leq \gamma_{0}|\gamma_{SR_{1}D}+\gamma_{SD}\leq \gamma_{0})\\ & & Pr(\gamma_{SR_{1}D}+\gamma_{SR_{2}D}+\gamma_{SR_{3}D}+\gamma_{SD}\leq \gamma_{0}|\gamma_{SR_{1}D}+\gamma_{SR_{2}D}+\gamma_{SD}\leq \gamma_{0}) \end{array}$$

Using the law of conditional probability, i.e., $P\left(A|B\right)=\frac{P(A\cap B)}{P(B)}$, we get:

(C3) $$\begin{array}{ccc} P_{out} & = & Pr\left(\gamma_{SD}\leq \gamma_{0}\right)\times \frac{Pr(\gamma_{SR_{1}D}+\gamma_{SD})}{Pr(\gamma_{SD}\leq \gamma_{0})}\times \frac{Pr(\gamma_{SR_{1}D}+\gamma_{SR_{2}D}+\gamma_{SD})}{Pr(\gamma_{SR_{1}D}+\gamma_{SD})} \end{array}$$

(C4) $$\begin{array}{ccc} & & \times \frac{Pr(\gamma_{SR_{1}D}+\gamma_{SR_{2}D}+\gamma_{SR_{3}D}+\gamma_{SD})}{Pr(\gamma_{SR_{1}D}+\gamma_{SR_{2}D}+\gamma_{SD})} \end{array}$$

(C5) $$\begin{array}{ccc} & = & Pr(\gamma_{SR_{1}D}+\gamma_{SR_{2}D}+\gamma_{SR_{3}D}+\gamma_{SD}\leq \gamma_{0}) \end{array}$$
